# Targeting circGDI2 disrupt HNRNPC-mediated mPORCN stabilization and enhance LGK-974 anti-tumor therapy in hepatocellular carcinoma

**DOI:** 10.1186/s12943-026-02638-1

**Published:** 2026-03-10

**Authors:** Yang Huang, Liangliang Xu, Linfeng Yang, Zhenru Wu, Li Li, Yu Dai, Junlong Dai, Ming Zhang, Pengsheng Yi, Li Jiang, Mingqing Xu

**Affiliations:** 1 Division of Liver Surgery, Department of General Surgery, West China Hospital, Sichuan University, Chengdu, Sichuan Province 610041 China; 2 Department of Hepato-Biliary-Pancrease II, Affiliated Hospital of North Sichuan Medical College, Nanchong, Sichuan Province 637000 China; 3 Institute of Clinical Pathology, Key Laboratory of Transplant Engineering and Immunology, NHC, West China Hospital, Sichuan University, Chengdu, Sichuan Province 610041 China; 4 Institute of Clinical Pathology, West China Hospital of Sichuan University, Chengdu, 610041 China; 5 Medical Data Analytics Center, Department of Medicine and Therapeutics, The Chinese University of Hong Kong, Hong Kong State Key Laboratory of Digestive Disease, Institute of Digestive Disease, The Chinese University of Hong Kong, Hong Kong, China

**Keywords:** Hepatocellular carcinoma, circRNA, RNA binding protein, Wnt/β-catenin pathway, LGK-974

## Abstract

**Background:**

The functions of circRNAs in hepatocellular carcinoma (HCC) till needs to be further elucidated.

**Methods:**

We assessed the biological functions of circGDI2 in vitro and in vivo by gain or loss of function experiments. Then, fuorescence in situ hybridization (FISH), immunofluorescence (IF), RNA pull-down, mass spectrometry, and RNA immunoprecipitation (RIP) were applied to explore the interaction between circGDI2 and heterogeneous nuclear ribonucleoprotein C (HNRNPC). Finally, in vitro and in vivo experiments were performed to explore the influence of circGDI2 on the anti-tumor activity of LGK-974, a porcupine O-acyltransferase (PORCN) inhibitor.

**Results:**

CircGDI2 was significantly overexpressed in HBV-related HCC, and its high expression was significantly associated with the growth and invasion characteristics of HCC. Functional experiments indicated that circGDI2 promoted the proliferation and metastasis of HCC cells both in vitro and in vivo. Mechanistic investigations revealed that circGDI2 physically binds to HNRNPC, facilitating its interaction with mPORCN, which stabilizes mRNA and promotes PORCN expression, thereby activating the Wnt signaling pathway and driving tumor proliferation and metastasis. Additionally, we found that the PORCN inhibitor LGK-974 effectively suppressed the proliferation and metastasis of HCC cells both in vitro and in vivo, and a series of experiments demonstrated that knocking down circGDI2 could enhance the antitumor effect of LGK-974, thereby maximizing the inhibition of HCC.

**Conclusion:**

CircGDI2 played a crucial role in the progression of HCC by interacting with HNRNPC to promote the Wnt signaling pathway. Meanwhile, LGK-974 can effectively inhibit HCC and targeting circGDI2 can enhance the antitumor effect of LGK-974.

**Supplementary Information:**

The online version contains supplementary material available at 10.1186/s12943-026-02638-1.

## Introduction

Primary liver cancer is currently the sixth most common malignancy in the world and ranks fourth in cancer-related mortality rates [1, 2]. Hepatocellular carcinoma (HCC) accounts for approximately 95% of all primary liver cancers, and 80% HCC cases are associated with HBV infection [2]. Despite significant advances in the diagnosis and treatment of HCC in recent years, only 30–40% of patients are eligible for curative treatments due to the asymptomatic nature of early-stage HCC and its high tendency for intrahepatic metastasis [2]. However, the five-year postoperative recurrence rate for HCC remains as high as 70% after radical resection [3], which is closely related to the intrinsic vascular invasion propensity of HBV-related HCC and the underlying presence of liver cirrhosis. Therefore, efficient early diagnostic biomarkers and potent therapeutic strategies for HCC are urgently needed.

CircRNAs are a novel class of non-coding RNAs (ncRNAs) discovered after microRNAs (miRNAs) and long non-coding RNAs (lncRNAs). They are abundant, covalently closed single-stranded transcripts without 5’ and 3’ ends, which confer them high stability and resistance to degradation [4]. As research progresses, circRNAs have been found to function as miRNA sponges, encode peptides, bind proteins to manipulate gene expression, and act as scaffolds for circRNA-protein complexes [5–9]. Studies have shown that circRNAs play crucial roles in biological processes such as tumor proliferation, migration, and invasion. For instance, circPDE5A, circPVT1, and circCCDC66 are closely associated with the malignant behavior of esophageal squamous cell carcinoma [10], gastric cancer [11], and colorectal cancer [12]. However, the expression profile of circRNAs in HCC tissues and adjacent normal tissues remain unclear, and further screening and identification of circRNAs closely related to the occurrence and progression of HCC are urgently needed.

Due to the prohibitive costs associated with sequencing, previous research typically identified tumor-associated circRNAs by conducting RNA-sequencing on a limited sample size [13–15]. Subsequently, a selection of the most differentially expressed circRNAs was subjected to validation using quantitative real-time polymerase chain reaction (qRT-PCR) on a more extensive sample scale. However, considering the high heterogeneity among tumors and the limited size of sequencing samples, the circular circRNAs ultimately selected by this method may not truly represent the most effective molecules in the initiation and progression of tumors. Furthermore, the potential biological functions of other differentially expressed circRNAs have also missed the opportunity for research. To explore circRNAs that play significant roles in HCC, we integrated the differentially expressed circRNAs from two sets of circRNA sequencing data, one focusing on liver cirrhosis during the initiation of HCC (including 6 samples), and the other comparing the differentially expressed circRNAs between HCC and matched non-tumor tissues (including 18 samples) [16, 17]. We focused on the circRNAs that were highly expressed in cirrhotic HCC cohort in the first dataset and HCC tissues in the second dataset. Then, a comprehensive investigation was conducted into the expression disparities, clinical significance, biological function, and underlying mechanism of these commonly differentially expressed circRNAs.

Through integrated analysis and large-sample size qRT-PCR validation, we finally identified circGDI2 (circBase ID: hsa_circ_0005379), derived from the GDI2 gene, is obviously upregulated in HBV-related HCC tissues when compared with matched non-tumor tissues. Functionally, circGDI2 efficiently promotes proliferation and metastasis in HCC both in vitro and in vivo. Mechanistically, circGDI2 could interact with HNRNPC, leading to enhance the stabilizing effect of HNRNPC on PORCN, which plays a key role in the Wnt/β-catenin signaling pathway. More importantly, knocking down of circGDI2 markedly strengthens anti-tumor effects of LGK-974 which is a PORCN inhibitor. These findings indicate that circGDI2 holds immense potential as a therapeutic target for HCC.

## Results

### CircGDI2 is highly expressed in HCC tissues, and high level of circGDI2 is related to poor survival outcome

**Fig. 1:**
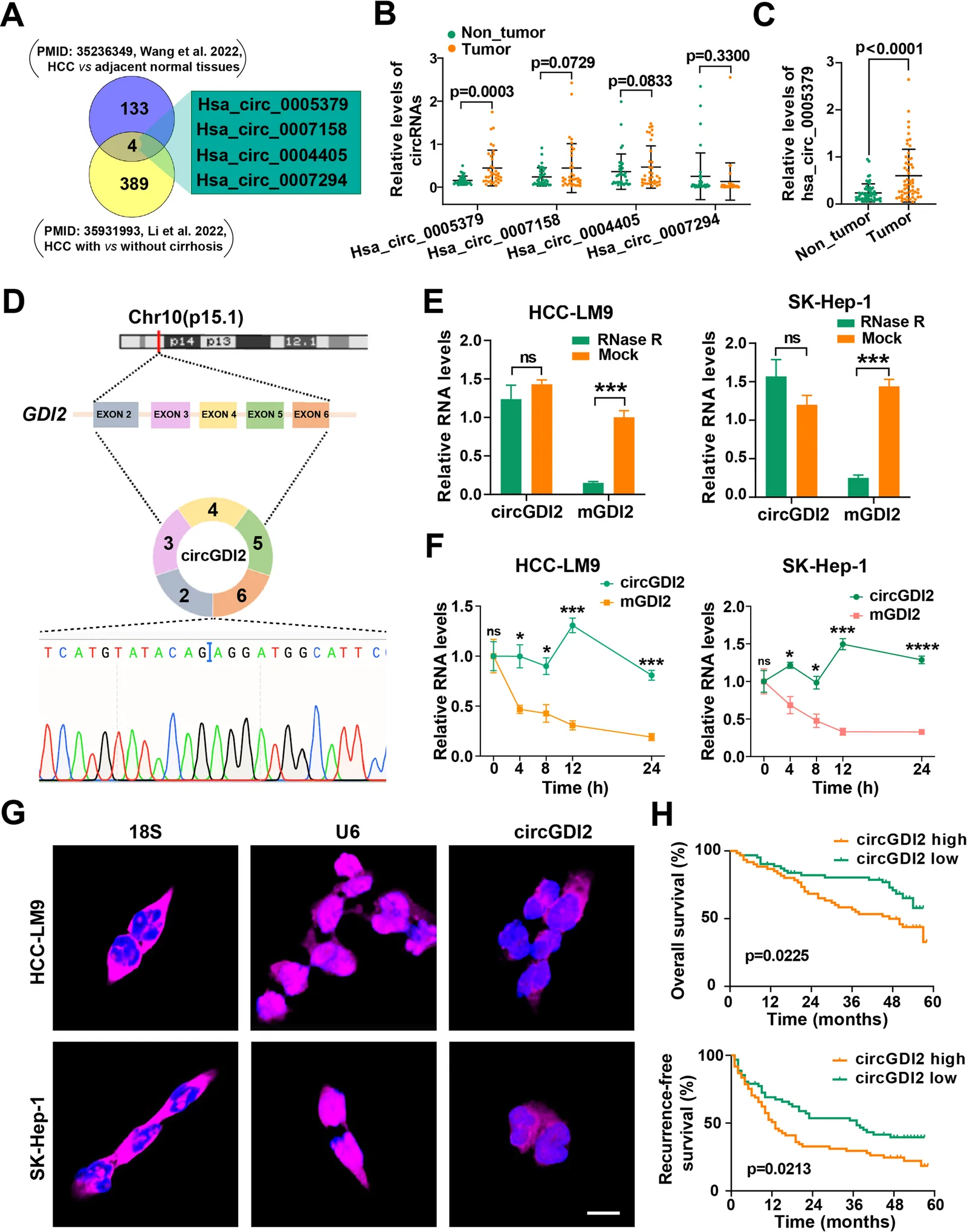
Identification and validation of circGDI2 and the relationship between circGDI2 and HCC patients. **A** Intersection between two RNA sequencing results to identify four common circRNAs. **B** Relative mRNA levels of four common circRNAs in 30 paired HBV-related HCC and adjacent normal tissues by qRT-PCR. **C** Relative mRNA levels of circGDI2 in 55 paired HBV-related HCC and adjacent normal tissues by qRT-PCR. **D** Schematic illustration of the genomic location and back splicing of circGDI2. Specific divergent primers were designed targeting the back-splice junction and qRT-PCR products were validated by Sanger Sequencing. **E** The stability of circGDI2 and mGDI2 was evaluated by qRT-PCR after RNase R management in HCC cells. **F** The stability of circGDI2 and mGDI2 was evaluated by qRT-PCR after Actinomycin D treatment in HCC cells. **G** The subcellular location of circGDI2 in HCC cells was investigated by FISH assay, scale bar = 20 μm. **H** Kaplan-Meier curves showing the OS (up) and RFS (down) of 123 HBV-related HCC patients. Patients were stratified by the median expression levels of circGDI2. *HCC* Hepatocellular carcinoma, *qRT-PCR* Quantitative reverse transcription polymerase chain reaction, *FISH* Fuorescence in situ hybridization, *OS* Overall survival, *RFS* Recurrence free-survival, *ns* No significance. Data are shown as mean ± SEM with p values by Mann-Whitney tests or log-rank for Kaplan-Meier curves. *: *P* < 0.05; ***: *P* < 0.001; ****: *P* < 0.0001

To screen circRNA that influence the progression of HCC, we focused exclusively on highly expressed circRNAs. In the first database, a total of 213 circRNAs were highly expressed in cirrhotic HCC samples, while in the second database, 76 circRNAs were highly expressed in HCC samples [16, 17]. We identified four common circRNAs that were upregulated in cancer tissues with a fold change > 1.5: hsa_circ_0005379、hsa_circ_0007158、hsa_circ_0004405、 hsa_circ_0007294 (Fig. 1A, Supporting Fig. S1A). To further validate the above results, we designed circRNA-specific divergent primers for these four circRNAs, validated by qRT-PCR and nucleic acid electrophoresis experiments (Supporting Fig. S1B-C). The Sanger sequencing results demonstrated that the PCR products amplified by these primers included the back-splicing junctions of the circRNAs (Supporting Fig. S1D). We firstly measured the expression levels of these four circRNAs in 30 pairs of HBV-related HCC tissues and adjacent normal tissues, revealing that only hsa_circ_0005379 (circGDI2) exhibited a significant difference in expression levels between tumor and adjacent normal tissues (*P* = 0.0003) (Fig. 1B). Subsequently, we expanded the clinical sample size to 55 pairs and found that the expression difference of hsa_circ_0005379 was further increased (*P* < 0.0001) (Fig. 1C). Similarly, Gene Expression Profiling Interactive Analysis (GEPIA) database indicated that the parental mRNA of circGDI2, *GDI2*, also had higher expression levels in HCC tissues compared to adjacent normal tissues (Supporting Fig. S1E). Consequently, we selected circGDI2 for further study.

To verify the circular characteristics of circGDI2. According to the circBase database and confirmed by Sanger Sequencing, circGDI2 is formed by the back-splicing of exons 2–6 of the GDI2 gene (Fig. 1D). To demonstrate that circGDI2 is a product of post-transcriptional back-splicing of GDI2, rather than an inherent component of the genome, we designed two sets of primers, including divergent primers for circular transcripts and convergent primers for linear transcripts. The two sets of primers were used to amplify the circular and linear transcripts of GDI2 in both cDNA and gDNA from HCC-LM9 and SK-Hep-1 cells. The circular transcripts were amplified by divergent primers in cDNA, but not in gDNA, while the linear transcripts could be amplified by convergent primers in both cDNA and gDNA. No product was amplified by divergent primers of GAPDH in cDNA and gDNA in the GAPDH negative control gene (Supporting Fig. S1F). Additionally, oligo(dT)18 and random hexamer primers were applied in reverse transcription experiments, significant decrease of circGDI2 and no significant change of mGDI2 expression in oligo(dT)18 group, indicating that circGDI2 had no poly-A tail (Supporting Fig. S1G). Then, we conducted an RNase R assay, which showed that after treatment with RNase R, the expression of circGDI2 remained stable, whereas the expression of mGDI2 was significantly decreased (Fig. 1E, Supporting Fig. S1H). Additionally, we treated HCC-LM9 and SK-hep-1 cells with actinomycin D, and the results indicated that circGDI2 has a longer half-life compared to mGDI2. Above two experiments suggested that circGDI2 possessed a stable circular structure (Fig. 1F). Finally, nuclear-cytoplasmic fractionation experiment and fluorescent in situ hybridization (FISH) assays showed that circGDI2 was predominantly located in the cytoplasm, where it may exert its biological function (Supporting Fig. S1I, Fig. 1G).

To explore the relationship between circGDI2 expression and clinicopathological features, we measured the expression of circGDI2 in 123 HBV-related HCC tissues. Based on the median expression level, the population were divided into low circGDI2 expression group (Low, 62 patients) and high circGDI2 expression group (High, 61 patients). As shown in Table S1, the High group had significantly more cases with serum alpha-fetoprotein (AFP) ≥ 400 ng/mL, macrovascular invasion, and microvascular invasion (MVI) compared to the Low group (*P* = 0.033, 0.012, and 0.012, respectively). Additionally, the degree of tumor differentiation and the BCLC stage were significantly worse in the High group (*P* = 0.003 and 0.013, respectively). Kaplan-Meier survival curves indicated that patients in the High group had shorter overall survival (OS, *P* = 0.0225) and recurrence-free survival (RFS, *P* = 0.0213) compared to the Low group (Fig. 1H). Similarly, GEPIA database indicated that the high mGDI2 was also correlated with poor OS and RFS (Supplemental Fig. S1J). Univariate analysis showed that serum AFP level, tumor size, macrovascular invasion, BCLC stage, MVI, tumor differentiation, satellite lesions, and circGDI2 level were correlated with OS; age, serum AFP level, tumor number, tumor size, tumor capsule invasion, macrovascular invasion, BCLC stage, MVI, satellite lesions, and circGDI2 level were correlated with RFS (Table S2). Multivariate regression analysis show that circGDI2 level, tumor differentiation, MVI, and tumor size were independent risk factors for OS, while circGDI2 level, MVI, and tumor size were regarded as independent risk factors for RFS (Supplemental Fig. S1K, Table S3). Accordingly, these results imply that circGDI2 may play essential roles in the deterioration of HCC.

### CircGDI2 promotes the proliferation and migration of HCC in vitro and its expression is regulated by EIF4A3

**Fig. 2:**
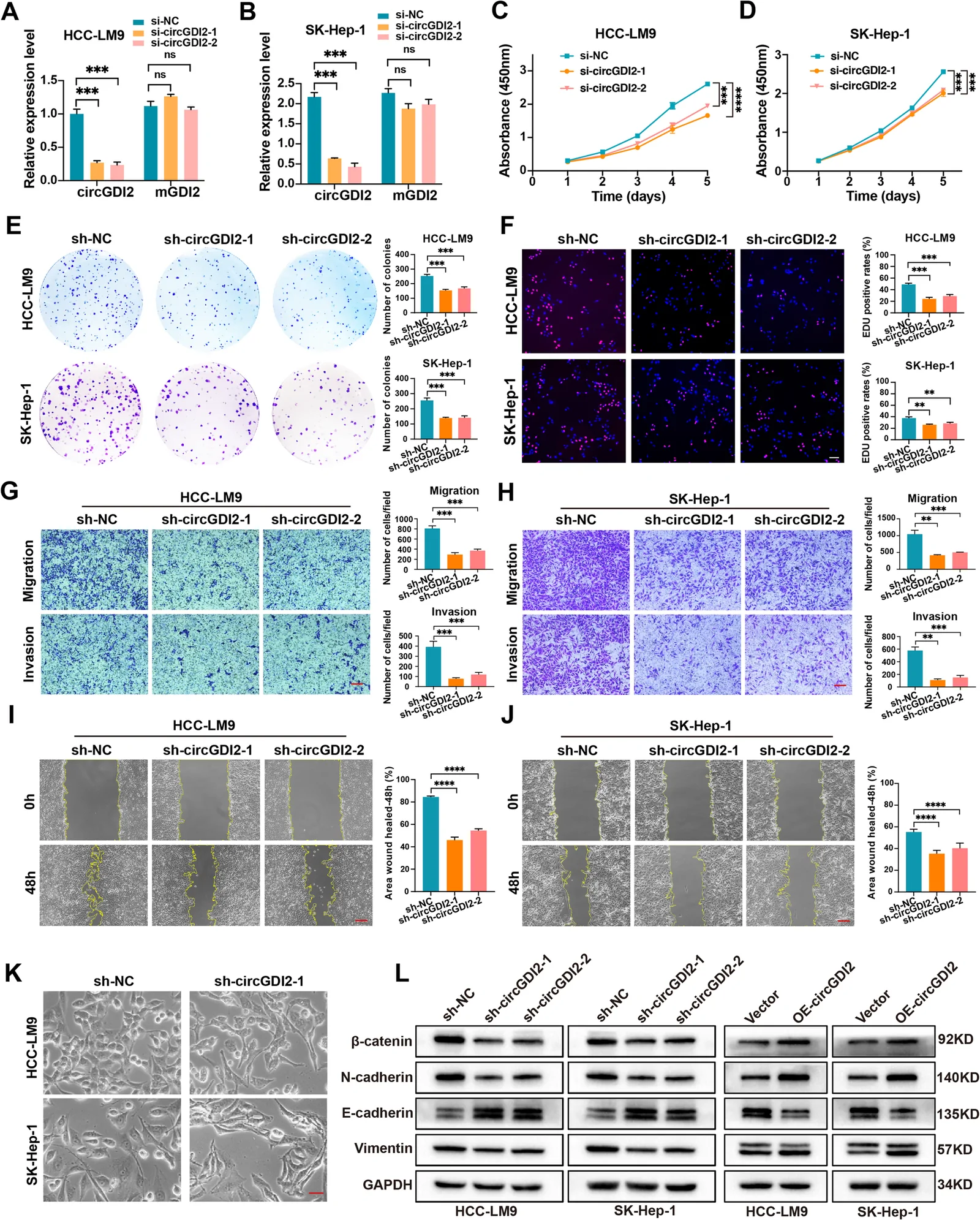
CircGDI2 promotes the proliferation and migration of HCC-LM9 and SK-Hep-1 cells in vitro. **A**-**B** The expression levels of circGDI2 and mGDI2 were measured by qRT-PCR after transfecting HCC cells with si-circGDI2. **C**-**D** CCK-8 assay was performed in HCC cells transfected with si-circGDI2. **E** Colony formation assay was conducted in HCC cells transfected with sh-circGDI2. **F** EdU assay was performed to detect the effects of circGDI2 on the capacity of DNA duplication of HCC cells transfected with sh-circGDI2. **G**-**H** Transwell assay was performed in HCC cells transfected with sh-circGDI2 to evaluate the migration capability. **I**-**J** Wound healing assay was performed in HCC cells transfected with sh-circGDI2. **K** The morphology of HCC-LM9 and SK-Hep-1 cells changed after knocking down circGDI2. **L** Western Blot showed the expression of EMT-related molecules in sh-circGDI2 and OE-circGDI2 HCC cells. *qRT-PCR* Quantitative reverse transcription polymerase chain reaction, *HCC* Hepatocellular carcinoma, *CCK-8* Cell Counting Kit-8, *EMT* Epithelial-Mesenchymal Transition, *OE* Overexpression, *ns* No significancy. Data are shown as mean ± SEM with p values by Mann-Whitney tests. **P: <0.01; ***P: <0.001; ****: *P* < 0.0001. Scale bars, 100 μm

To validate the biological functions of circGDI2 in HCC cells, we first measured the expression levels of circGDI2 in seven HCC cell lines and selected HCC-LM9 and SK-Hep-1 cells as the tool cells for this study because of their high and stable expression levels of circGDI2 (Supporting Fig. S2A). Subsequently, we transfected HCC-LM9 and SK-Hep-1 cells with small interfering RNA (siRNA) targeting the circGDI2 back-splicing junction, qRT-PCR results showed that the constructed siRNA effectively knocked down circGDI2 without significantly affecting mGDI2 expression (Fig. 2A-B). The CCK-8 assay demonstrated that silencing circGDI2 significantly inhibited the proliferation of HCC cells (Fig. 2C-D, Supporting Fig. S3A). These results preliminarily suggested that circGDI2 had the function in facilitating the malignant progression of HCC. To complete the subsequent cellular functional assays, circGDI2 was stably knocked down and overexpressed through lentivirus infection in HCC-LM9, SK-Hep-1, Huh7 and Hep3B cells, and the efficiency was verified by qRT-PCR in HCC-LM9 and SK-Hep-1 cells (Supporting Fig. S2B-C).

Firstly, we performed the clone formation and EdU assays to measure the effect of circGDI2 on cell proliferation. These results demonstrated that circGDI2 knockdown suppressed the proliferation of HCC cells (Fig. 2E-F, Supporting Fig. S3B-C). As expected, overexpression of circGDI2 produced the opposite effects (Supporting Fig. S2D-F, Supporting Fig. S3F-H). Consistently, transwell assay and wound healing assay revealed that circGDI2 knockdown impeded migration ability, while overexpression of circGDI2 promoted migration ability (Fig. 2G-J and Supporting Fig. S3D-E; Supporting Fig. S2G-J and Supporting Fig. S3I-K, respectively). These findings indicated the oncogenic role of circGDI2 in HCC cells. Interestingly, we observed significant morphological changes in HCC-LM9 and SK-Hep-1 cells after circGDI2 knockdown. HCC-LM9 cells tended to aggregate and displayed a skeletal morphology, while SK-Hep-1 cells became flattened and elongated, also exhibiting a more skeletal structure (Fig. 2K). Meanwhile, the expression level of multiple Epithelial-Mesenchymal Transition (EMT)-related proteins, namely β-catenin, E-cadherin, N-cadherin and Vimentin, were also obviously changed along with the knockdown and overexpression of circGDI2 (Fig. 2L, Supporting Fig. S3L). These evidences suggested that circGDI2 may exert a significant regulatory effect on the EMT related pathway.

RNA-binding proteins (RBP) regulate the biogenesis of circRNAs by adhering to up or downstream introns flanking the back-splicing sites [18, 19]. Using the online CircInteractome tool (https://circinteractome.irp.nia.nih.gov/), we found three potential RBPs of circGDI2 pre-mRNA (Supporting Fig. S4A). Among these, EIF4A3, an RNA helicase primarily involved in RNA splicing and metabolic processes [20], plays a critical role in various types of tumors by promoting the biogenesis of circRNAs [21–23]. To validate the relationship between EIF4A3 and circGDI2, specific siRNAs were designed. The qRT-PCR results demonstrated that EIF4A3 depletion significantly decreased circGDI2 expression in HCC cells (Supporting Fig. S4B). GEPIA database indicated that the high EIF4A3 was also correlated with poor OS (*p* = 0.00049) and RFS (*p* = 0.057) in HCC patients (Supporting Fig. S4C-D). Pearson correlation analysis revealed that the expression of EIF4A3 in HCC tissues was positively correlated with circGDI2 (Supporting Fig. S4E). Taken together, these results preliminarily suggested that EIF4A3 facilitate the biogenesis of circGDI2.

### CircGDI2 promotes the growth and metastasis of HCC in vivo

**Fig. 3:**
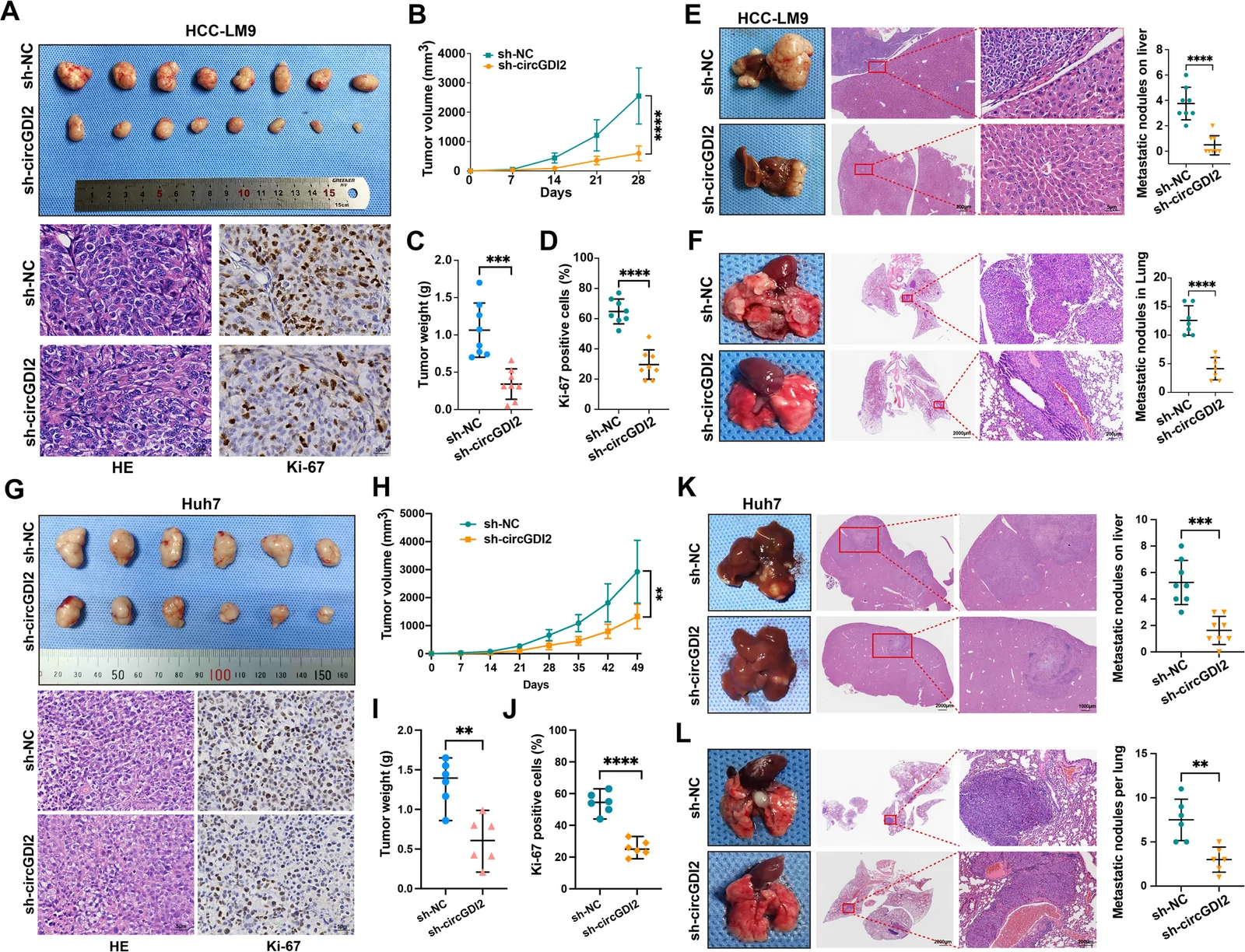
CircGDI2 facilitates the tumor growth and metastasis of HCC in vivo. **A** Subcutaneous xenograft tumors dissected from nude mice inoculated with indicated circGDI2-knockdown HCC-LM9 cell. Representative HE and Ki-67 staining images of subcutaneous xenograft tumors. **B**-**C** Volume (up) and weight (down) of subcutaneous xenograft tumors from figure A. **D** The proportion of Ki-67 positive cells of subcutaneous xenograft tumors was indicated in the dot graph. **E** Representative xenograft tumors and HE staining of metastatic nodules of liver orthotopic-implantation models inoculated with indicated circGDI2-knockdown HCC-LM9 cells. **F** Representative images of lung tumors and HE staining of metastatic nodules of lung metastasis model inoculated with indicated circGDI2-knockdown HCC-LM9 cells. The number of metastatic foci formed in the lungs was indicated in the dot graph. **G** Subcutaneous xenograft tumors inoculated with indicated circGDI2-knockdown Huh7 cell and representative HE and Ki-67 staining images. **H**-**I** Volume (up) and weight (down) of subcutaneous xenograft tumors from figure G. **J** The proportion of Ki-67 positive cells of subcutaneous xenograft tumors was indicated in the dot graph. **K** Representative xenograft tumors and HE staining of metastatic nodules of liver orthotopic-implantation models. The number of metastatic foci formed in the livers was indicated in the dot graph. **L** Representative images of lung tumors and HE staining of metastatic nodules of lung metastasis model. The number of metastatic foci formed in the lungs was indicated in the dot graph. *HCC* Hepatocellular carcinoma, *HE* Hematoxylin-eosin. Data are shown as mean ± SEM with p values by Mann-Whitney tests. ***P < 0.001, ****P < 0.0001

To validate whether circGDI2 still promotes malignant progression of HCC in a complex in vivo environment. We firstly subcutaneously inoculated stable HCC-LM9 and Huh7 cells transfected with sh-circGDI2 and the corresponding control sh-NC into nude mice. In the xenograft model, the tumor growth rate in the circGDI2 knockout group was slower, and the tumor volume was smaller compared to the control group. Immunohistochemistry (IHC) staining revealed weaker Ki-67 levels in the circGDI2 knockout group (Fig. 3A-D, G-J). These results suggest that circGDI2 promotes HCC proliferation in vivo.

Next, we utilized the HCC-LM9 and Huh7 cells transfected with sh-circGDI2 and the corresponding control sh-NC to establish the liver orthotopic-implantation models and lung metastasis models. These representative results showed that circGDI2 knockout group showed smaller tumor volume and fewer metastatic foci in both liver and lung. Haematoxylin eosin (HE) staining confirmed its inhibitory role in tumor metastasis when knocking down circGDI2 (Fig. 3E-F, K-L). In summary, the above data indicated that circGDI2 substantially inhibited the growth and metastasis of HCC cell both in vivo.

### CircGDI2 activates Wnt/β-catenin signaling pathway

**Fig. 4:**
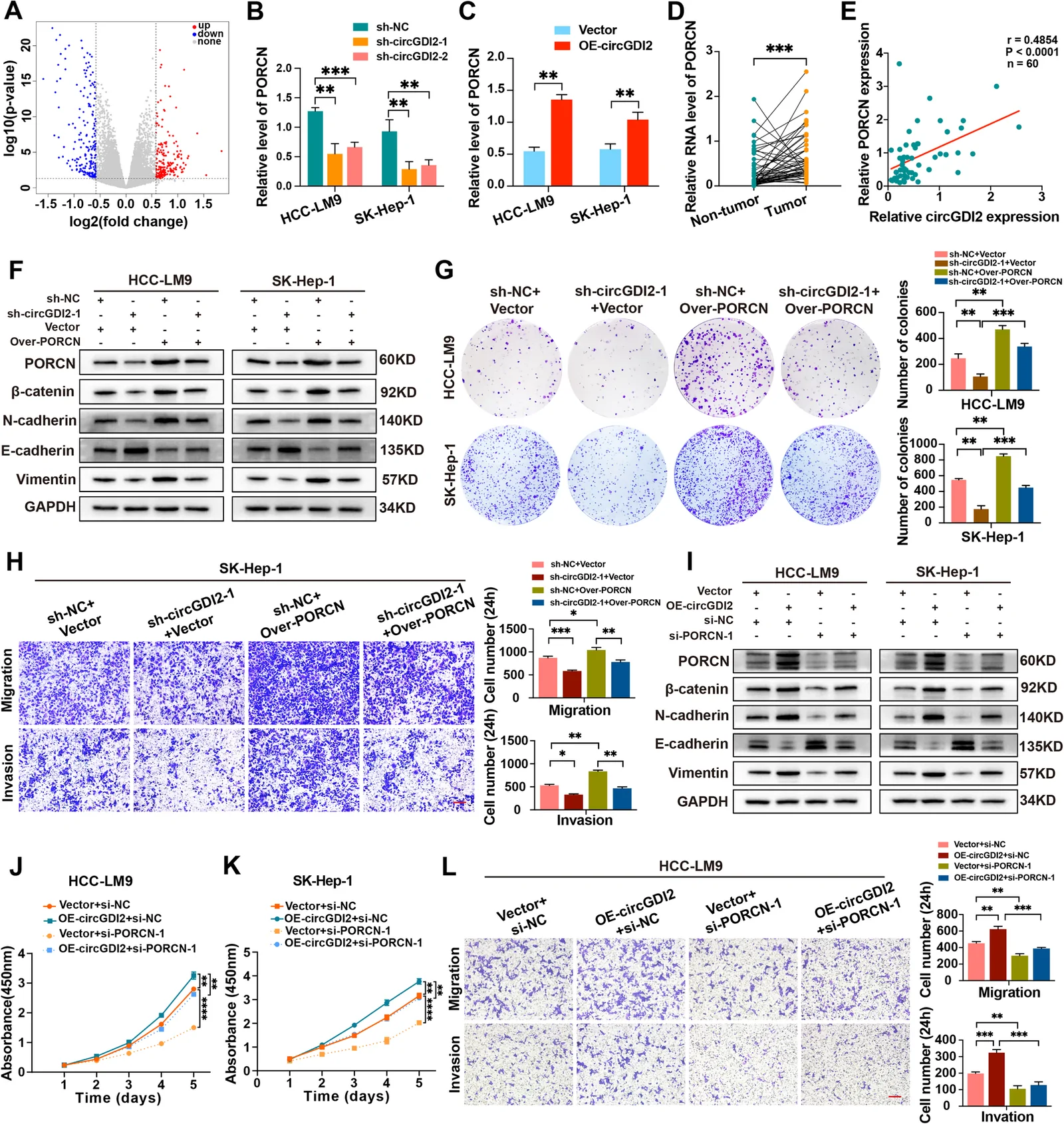
CircGDI2 inactivates Wnt/β-catenin signaling pathway. **A** Volcano plot representing sequencing results of HCC-LM9 cells transfected with si-NC and si-circGDI2. **B**-**C** qRT-PCR detected the expression of PORCN in HCC cells transfected with sh-circGDI2 and OE-circGDI2. **D** Relative levels of PORCN in 55 paired HBV-related HCC and adjacent normal tissues by qRT-PCR. **E** Pearson’s correlation test was performed to clarify the correlation between PORCN and circGDI2 levels of tumor tissues from 60 HBV-related HCC patients. **F** Western Blot for the expression of EMT-related molecules in the indicated HCC cells. **G** Colony formation assay showed the proliferation capacity of the indicated HCC cells. **H** Transwell assay showed the migration and invasion capacity of the indicated SK-Hep-1 cell. **I** Western Blot analysis for the expression of EMT-related molecules in the indicated cells. **J**-**K** CCK8 assay showed the proliferation capacity of the indicated HCC cells. **L** Transwell assay showed the migration and invasion capacity of the indicated HCC-LM9 cell. *HCC* Hepatocellular carcinoma, *OE* Overexpression, *qRT-PCR* Quantitative reverse transcription polymerase chain reaction, *EMT* Epithelial-Mesenchymal Transition, *CCK-8* Cell Counting Kit-8. Data are shown as mean ± SEM with p values by Mann-Whitney tests. *P: <0.05; **P: <0.01; ***P: <0.001; ****: *P* < 0.0001. Scale bars, 100 μm

To illuminate the molecular mechanisms by which circGDI2 promote HCC cell proliferation and metastasis, we performed RNA-seq to identify potential regulatory genes affected by circGDI2 knockdown. A total of 410 genes were found to be differentially expressed, including 200 up-regulated and 210 down-regulated genes (Fig. 4A, Supporting Fig. S5A, Table S4). Through systematically retrieving the biological functions of these differentially expressed genes, PORCN has caught our attention (logFC = 1.29; *p* = 0.000057). It is a member of the membrane-bound O-acyltransferase superfamily, capable of palmitoylating Wnt ligands to initiate the Wnt pathway and is highly specific to this pathway [24]. It has been reported that the Wnt signaling pathway is closely related to the occurrence of EMT [25]. Considering our previous findings that circGDI2 has a potential in regulating the EMT related pathway, we hypothesized that circGDI2 may promote HCC growth and metastasis through the PORCN/Wnt pathway. To demonstrate the regulatory effect of circGDI2 on PORCN, we examined the PORCN levels in HCC cell lines with stable circGDI2 regulation, both the RNA and protein levels of PORCN were positively regulated by circGDI2 (Fig. 4B-C, Supporting Fig. S5B-C), these results were in consistent with the RNA-sequencing. Additionally, qRT-PCR results showed that PORCN levels in HBV-related HCC tissues were significantly higher than that in adjacent normal tissues (*P* < 0.001, Fig. 4D). Moreover, correlation analysis of PORCN and circGDI2 expression levels revealed a significant positive correlation in HBV-related HCC tissues (*r* = 0.4854, *P* < 0.0001, Fig. 4E). β-catenin is a key regulator in the Wnt/β-catenin signaling pathway. Therefore, to elucidate whether circGDI2 influences the alteration in β-catenin’s subcellular localization. Nuclear and cytoplasmic fractionation assays showed that circGDI2 knockdown significantly reduced the β-catenin protein levels of in the nuclear of HCC cells (Supporting Fig. S5D). These results demonstrated that circGDI2 activates the Wnt/β-catenin pathway.

To elucidate the regulatory relationship between circGDI2 and PORCN/Wnt pathway, firstly, the expression of PORCN was artificially regulated through lentivirus infection or special siRNAs, and the efficiency was verified by qRT-PCR and Western Blot (Supporting Fig. S6A-C). Subsequently, A series of in vitro experiments showed that PORCN overexpression could effectively promote the proliferation and metastasis of HCC cells (Supporting Fig. S6D-J), and knocking down PORCN achieved inverse results (Supporting Fig. S7A-C). More importantly, we found that over-PORCN could reversely rescue the suppressive effect of sh-circGDI2 on the proliferation, metastasis, and the expression of EMT related proteins in HCC cells (Fig. 4F-H, Supporting Fig. S7D-G). Meanwhile, si-PORCN could weaken the enhanced effect of over-circGDI2 on the proliferation, metastasis (Fig. 4I-L, Supporting Fig. S7H-K). These results collectively indicated that PORCN is a pivotal downstream target of circGDI2 and efficiently mediated the biological functions of circGDI2.

### CircGDI2 promotes the proliferation and migration of HCC in vitro through interacting with HNRNPC

**Fig. 5:**
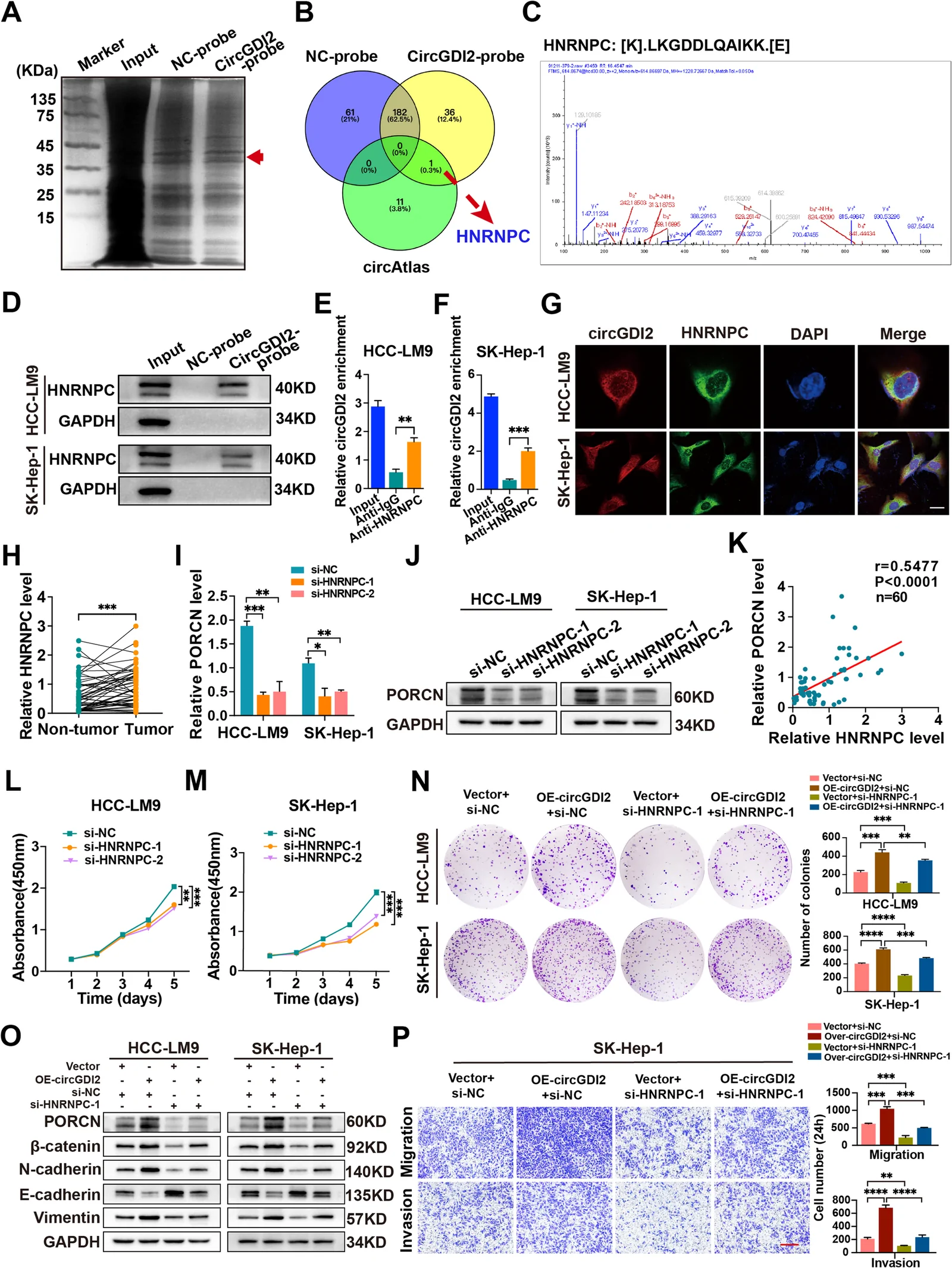
CircGDI2 interacts with HNRNPC to mediate PORCN/Wnt signaling pathway activation. **A** CircGDI2 probe and control probe were applied for RNA pull-down assay in HCC-LM9 cells, followed by Coomassie brilliant staining. A specific band, indicated by the arrow, appeared at approximately 40kD. **B** HNRNPC was identified by intersecting the MS analysis results with the predictions from circAltas. **C** Peak map of HNRNPC acquired from the MS analysis. **D** Protein pulled down by circGDI2 probe with HNRNPC antibody was detected by Western Blot. **E**-**F** RIP assay was performed using HNRNPC or IgG antibodies in HCC cells. **G** The localization of circGDI2 and HNRNPC were detected by FISH assay and immunofluorescence assays, scale bar = 25 μm. **H** Relative mRNA levels of HNRNPC were detected in 55 paired HBV-related HCC and adjacent normal tissues by qRT-PCR. **I**-**J** The expression levels of PORCN were measured by qRT-PCR and Western Blot after transfecting HCC cells with si-HNRNPC. **K** Pearson’s correlation test was performed to clarify the correlation between HNRNPC and PORCN levels of 60 HBV-related HCC tissues. **L**-**M** CCK-8 assay was performed in HCC cells transfected with si-HNRNPC. **N** Colony formation assay showed the proliferation capacity of the indicated HCC cells. **O** Western Blot results for the expression of EMT-related molecules in the indicated HCC cells. **P** Transwell assay showed the migration and invasion capacity of the indicated SK-Hep-1 cell. *MS* Mass spectrometry, *RIP* RNA Immunoprecipitation; RNA immunoprecipitation, *HCC* Hepatocellular carcinoma, *FISH* Fuorescence in situ hybridization, *qRT-PCR* Quantitative reverse transcription polymerase chain reaction, *CCK-8* Cell Counting Kit-8, *EMT* Epithelial-Mesenchymal Transition. Data are shown as mean ± SEM with p values by Mann-Whitney tests. **P: <0.01; ***P: <0.001; ****: *P* < 0.0001. Scale bars, 100 μm

CircRNAs function as miRNA sponges, protein decoys or the template to encode small peptides [26]. To elucidate how circGDI2 regulated the expression of PORCN, we first predicted all potential miRNAs that could bind to circGDI2 using CircInteractome (https://circinteractome.nia.nih.gov/). Among them, 11 miRNAs had a context+ score percentile greater than 90. Subsequently, we conducted RNA immunoprecipitation (RIP) assay using AGO2 antibody to mediate the interaction between circGDI2 and miRNAs. However, circGDI2 was not enriched in HCC-LM9 and SK-Hep-1 cells (Supporting Fig. S8A). Additionally, circRNADb (http://reprod.njmu.edu.cn/circrnadb) indicated that circGDI2 has a low coding potential (Supporting Fig. S8B). Subsequently, we presumed that circGDI2 might interact with protein. To verify this hypothesis, biotinylated probe that targets the back-spliced site of circGDI2 and NC probe were used for RNA pull-down in HCC-LM9 and SK-Hep-1 lysates, followed by Coomassie brilliant blue staining and mass spectrometry (MS) analysis. A specific band at approximately 40 kD was observed in the circGDI2 probe group (Fig. 5A). Furthermore, based on the MS results (Table S5), using Unique Peptides > 2 as the criterion and excluding molecules common to the NC group, 40 molecules were selected. Ultimately, we intersected these results with 12 RNA binding proteins (RBPs) potentially binding circGDI2 predicted from circAltas (http://circatlas.biols.ac.cn) (Table S6), resulting in the identification of a single common molecule: HNRNPC (Fig. 5B-C). We next confirmed the interaction between circGDI2 and HNRNPC in HCC-LM9 and SK-Hep-1 cells by RNA pull-down and RIP assays (Fig. 5D-F). The co-localization of circGDI2 and HNRNPC was further visualized by simultaneously FISH and immunofluorescence (IF) assays (Fig. 5G).

HNRNPC is an important member of the heterogeneous nuclear ribonucleoprotein family (hnRNPs), which is closely associated with tumor development. To test its biological function in HCC, clinically, we detected a significantly higher expression of HNRNPC in HCC tissues compared to adjacent normal tissues using qRT-PCR (Fig. 5H). HNRNPC depletion significantly decreased PORCN expression in HCC cells and HNRNPC had a positive relationship with PORCN expression (Fig. 5I-K). Based on the expression levels of HNRNPC in 123 HBV-related HCC specimens, we classified them into low expression group (Low, 62 patients) and high expression group (High, 61 patients) using the median value as the cutoff. Kaplan-Meier survival curves indicated that patients with high HNRNPC expression had significantly shorter OS and RFS compared to those with low expression (*P* = 0.0178, *P* = 0.0007, respectively) (Supporting Fig. S8C-D). To confirm whether the effect of circGDI2/HNRNPC complex on promoting HCC progression, we conducted a series of functional and rescue experiments. We first knocked down HNRNPC using siRNA and validated this knockdown with qRT-PCR and Western Blot (Supporting Fig. S8E-F). CCK-8 assays showed that knocking down HNRNPC inhibited the proliferation of HCC cells (Fig. 5L-M). Furthermore, clone formation assay, Western Blot, and transwell assays demonstrated that overexpression of circGDI2 promoted proliferation and migration, which could be abrogated by interfering with HNRNPC (Fig. 5N-P, Supporting Fig. S8G-K).

### CircGDI2 enhances the stabilizing effect of HNRNPC on *PORCN*

**Fig. 6:**
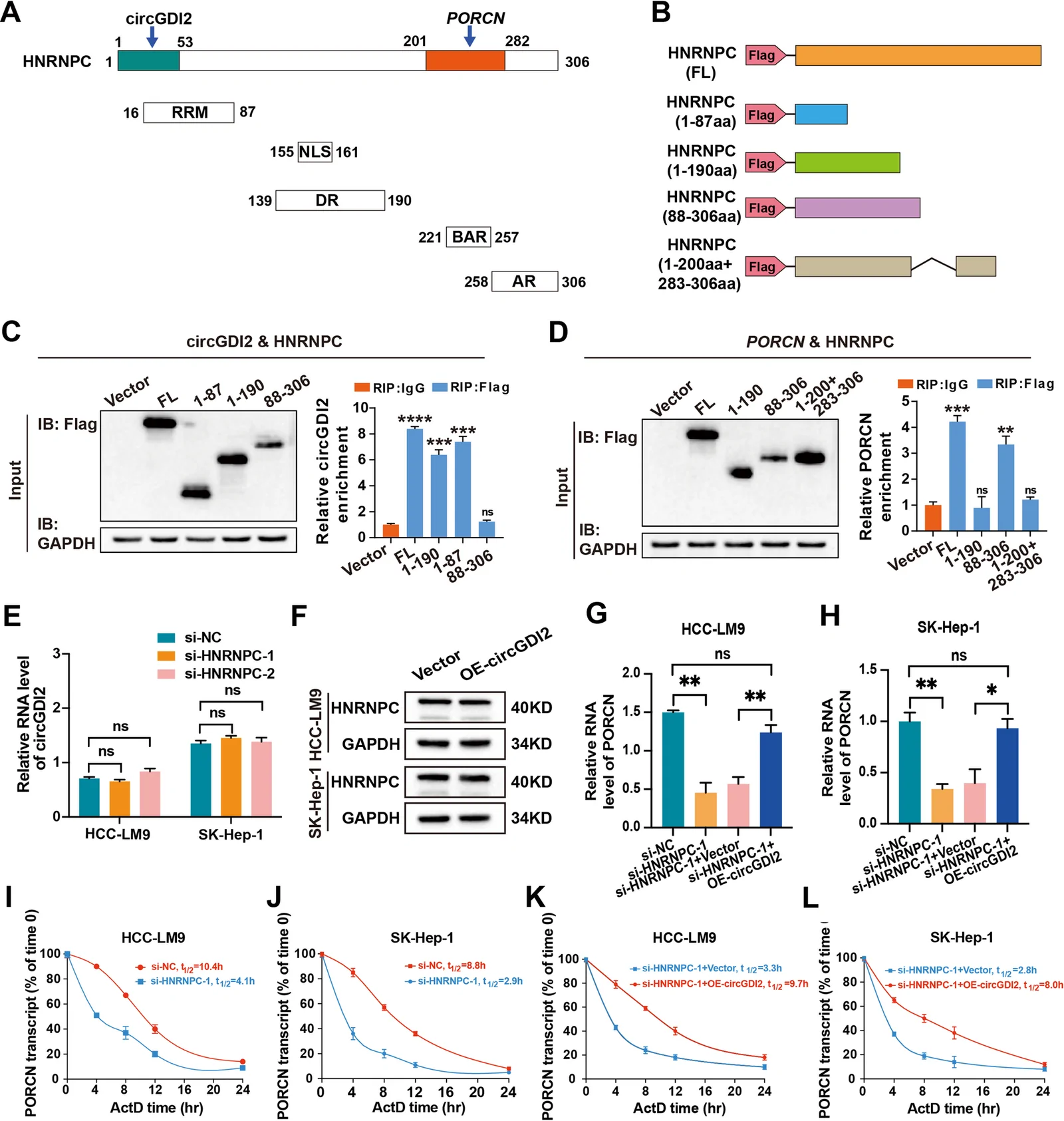
CircGDI2 promotes Wnt pathway by interacting with HNRNPC to stabilize mPORCN. **A** Prediction of RNA-protein interaction among circGDI2, HNRNPC and mPORCN using the catRAPID algorithm. **B** The diagrams of domain structure of HNRNPC and Flag-tagged HNRNPC truncations. **C**-**D** Western Blot showed the expression of full length or HNRNPC truncations from lysates of HEK293T cells transfected with the indicated vectors, and RIP assay was performed to detect the enrichment of circGDI2 in 293T cells transfected with full-length and truncated Flag-tagged truncations. **E** qRT-PCR texted the expression levels of circGDI2 in HCC cells transfected with si-HNRNPC. **F** Western Blot texted the expression levels of HNRNPC in HCC cells transfected with OE-circGDI2. **G**-**H** qRT-PCR texted the expression levels of PORCN in HCC cells transfected with si-PORCN alone or si-HNRNPC combined with OE-circGDI2. **I**-**J** Knocking down HNRNPC shortened the half-life of PORCN after actinomycin D treatment. **K**-**L** The effect of knocking down HNRNPC in figure (**I**-**J**) could be rescued by OE-circGDI2 in HCC cells. *RRM* RNA Recognition Motif, *NLS* Nuclear Localization Signal, *DR* Dipeptide Repeat, *BAR* Bin/Amphiphysin/Rvs domain, *AR* Accessory Regions, *RIP* RNA immunoprecipitation, *qRT-PCR* Quantitative reverse transcription polymerase chain reaction, *HCC* Hepatocellular carcinoma, *OE* Overexpression, *ns* No significance. Data are shown as mean ± SEM with p values by Mann-Whitney tests. *P: <0.05; **P: <0.01; ***P: <0.001; ****: *P* < 0.0001

To further delineate the regulatory relationships among circGDI2, HNRNPC, and PORCN. We used catRAPID algorithm (http://s.tartaglialab.com/page/catrapid_group) for RNA-protein and RNA-RNA interaction prediction. Interestingly, we predicted distinct binding regions of HNRNPC with circGDI2 and mPORCN (Table S7 and Table S8, respectively). However, there is no direct binding relationship between circGDI2 and PORCN. Subsequently, according to predicted binding site, a series of Flag-tagged HNRNPC deletion mutants were designed to confirm the interaction among circGDI2, HNRNPC, and *PORCN* (Fig. 6A-B). RIP assay based on Flag-antibody revealed that HNRNPC (1-87aa) was the core region to bind circGDI2, and HNRNPC (201-282aa) was the indispensable domain for interacting with *PORCN* (Fig. 6C-D). Next, we overexpressed the 1-87aa fragment of HNRNPC in both HCC-LM9 and Huh7 cell lines. This overexpression led to a prolonged half-life and increased stability of mPORCN. These findings provide further validation for the interaction between circGDI2 and HNRNPC (Supporting Fig. S9A).

Given HNRNPC is an RBP with functions in stabilizing RNA and promoting RNA metabolism and transport, we measured the expression of circGDI2 and PORCN after HNRNPC knockdown and also measured the expression of HNRNPC after circGDI2 overexpression in HCC cells by qRT-PCR and Western Blot. The results showed that the expression levels of circGDI2 and HNRNPC do not change in response to alterations in each other’s expression (Fig. 6E-F). Interestingly enough, knockdown of HNRNPC significantly inhibited the expression of PORCN, and the effect could be rescued by overexpressing circGDI2 (Fig. 6G-H). Considering we have demonstrated that circGDI2 can positively regulate the expression of PORCN, we speculate that circGDI2 may strengthen the stability effect of HNRNPC on PORCN. To validate this hypothesis, we knocked down HNRNPC in HCC cells and added actinomycin D, and qRT-PCR was used to measure PORCN levels at various time points. The results showed that the half-life of the PORCN in the si-HNRNPC group was shorter than that in the control group after the addition of actinomycin D in HCC cells. However, in HCC cells with overexpressed circGDI2 and simultaneous knockdown of HNRNPC, the half-life of the PORCN was prolonged compared to the group with HNRNPC knockdown alone after the addition of actinomycin D (Fig. 6I-L). To further investigate the regulatory effects of circGDI2 in vivo, we established xenograft tumor models in nude mice using HCC cells overexpressing circGDI2. The results demonstrated that circGDI2 promoted PORCN expression to accelerate tumor growth (Supporting Fig. S9B-D) but had no significant effect on HNRNPC expression (Supporting Fig. S9E-G). These experiments suggested that circGDI2 regulated the expression of PORCN by enhancing the property of HNRNPC in stabilizing RNA expression.

### CircGDI2 serves as a promising therapeutic molecule and enhances anti-tumor effects of LGK-974 both in vitro and vivo

**Fig. 7:**
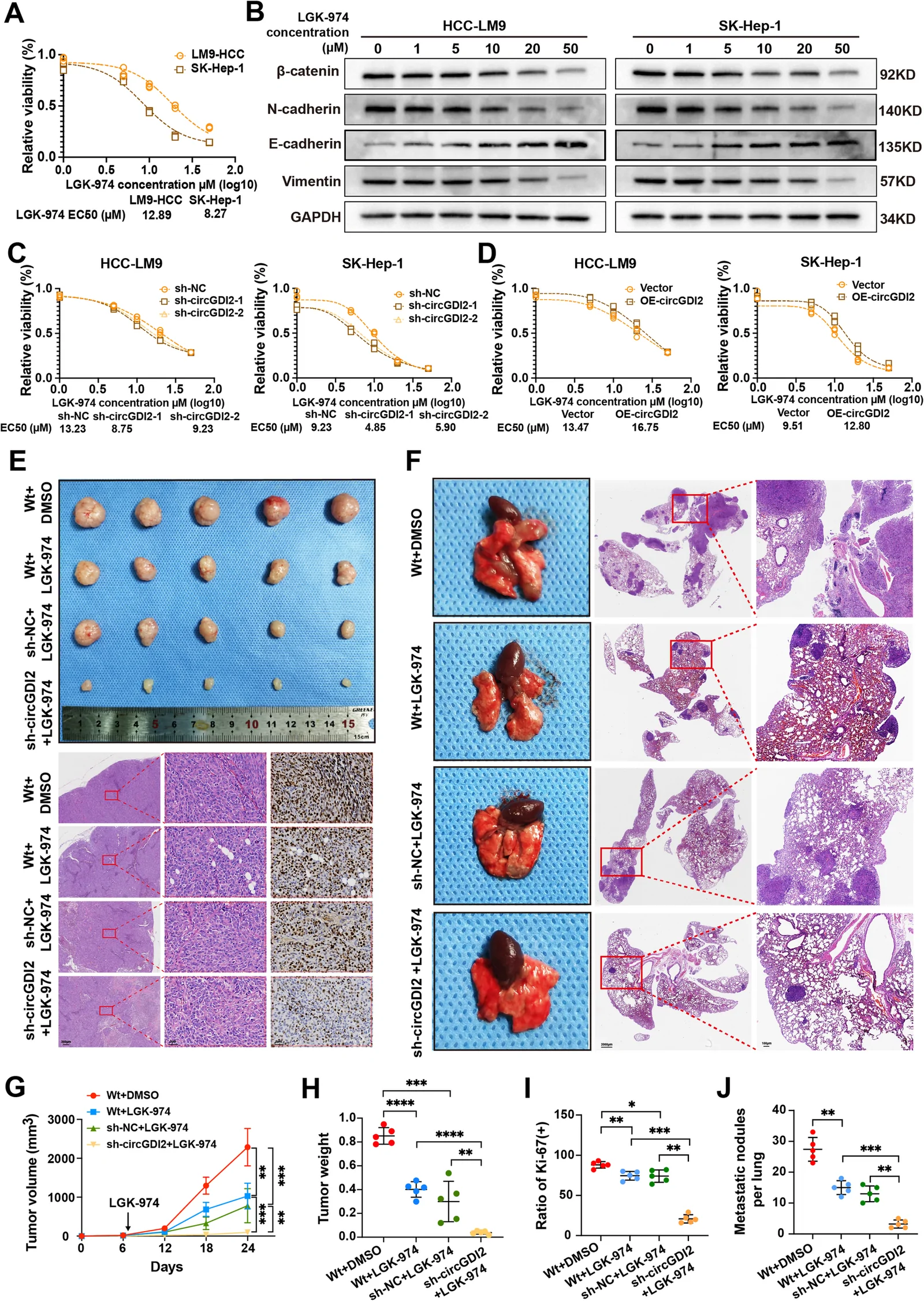
CircGDI2 alleviates anti-tumor effects of in vitro and in vivo. **A** CCK-8 assay was used to assess the cell viability on the third day in wild-type HCC-LM9 and SK-Hep-1 cells administered with different concentrations of LGK-974. **B** Western Blot texted for the expression of EMT-related molecules in HCC cells treated with the different concentration of LGK-974. **C**-**D** CCK-8 assay was used to assess the cell viability in knocking down and overexpressing circGDI2 HCC cells administered with different concentrations of LGK-974. **E** Representative images of subcutaneous xenografts. Mice were then dosed orally with LGK-974 at 5 mg/kg or DMSO twice daily after inoculating indicated HCC-LM9 for one week. Subcutaneous tumors were excised from node mice at day 17 after LGK-974 treatment, and representative HE and Ki-67 staining images of subcutaneous xenograft tumors. **F** Representative images of lung metastasis models. Mice were treated with LGK-974 at 5 mg/kg or DMSO twice daily after inoculating indicated HCC-LM9 for two months, and representative HE staining images of lung metastasis nodules. **G** Tumors volume of subcutaneous xenografts was indicated in the line graph. **H** Tumors weight of subcutaneous xenografts was indicated in the dot graph. **I** The proportion of Ki-67 positive cells of subcutaneous xenograft tumors was indicated in the dot graph. **J** The number of metastatic foci formed in the lungs was indicated in the dot graph. *EMT* Epithelial-Mesenchymal Transition, *HCC* Hepatocellular carcinoma, *CCK-8* Cell Counting Kit-8, *HE* Hematoxylin-eosin, *DMSO* Dimethyl Sulfoxide, *HE* Hematoxylin-eosin. Data are shown as mean ± SEM with p values by Mann-Whitney tests. *P < 0.05, **P < 0.01, ***P < 0.001, ****P < 0.0001

PORCN is a key molecule in the Wnt signaling pathway. LGK-974, a well-established inhibitor of PORCN, has been demonstrated to effectively suppress the Wnt signaling pathway in various tumors [27]. To validate LGK-974 also has an inhibitory effect on HCC cells, initially, we set up a drug concentration gradient (0, 5, 10, 20, 50 µM) in HCC-LM9 and SK-Hep-1 cells. On the third day, the EC50 values for HCC-LM9 and SK-Hep-1 were detected by the CCK8 assay, yielding values of 12.89 µM and 8.27µM, respectively (Fig. 7A). Concurrently, on the third day, protein immunoblotting revealed that as the concentration of LGK-974 increased, the molecular levels of β-catenin, N-cadherin, and Vimentin gradually decreased, the level of E-cadherin gradually increased (Fig. 7B). Functionally, clone formation assay, transwell assays, and Western Blot demonstrated that LGK-974 inhibited the proliferation and migration of HCC cells, these effects could be rescued by overexpressing circGDI2 (Supporting Fig. S10A-G). Given that circGDI2 can regulate the expression of *PORCN*, we hypothesized that regulating circGDI2 could potentiate the anti-tumor effect of LGK-974. To validate this hypothesis, we knocked down circGDI2 and found that it reduced the EC50 value of LGK-974 in HCC cells, and overexpressed circGDI2 significantly increased the EC50 value of LGK-974 in HCC cells (Fig. 7C-D). These results suggested that HCC cells can be effectively inhibited by PORCN inhibitor LGK-974, and HCC cells with low levels of circGDI2 expression became more sensitive to LGK-974.

To validate the combined therapeutic effect of knockdown of circGDI2 and LGK-974. We designed the experiment in vivo that included four groups: Wt+DMSO, Wt + LGK-974, sh-NC + LGK-974, and sh-circGDI2 + LGK-974. Subcutaneous xenograft models revealed that administration of LGK-974 alone could suppress tumor growth, and this inhibitory effect was remarkedly enhanced after the combination of circGDI2 knockdown (Fig. 7E, G-H, Supporting Fig. S11A-B). HE staining results also showed that the sh-circGDI2 + LGK-974 group had the lowest proportion of Ki-67 positive cell (Fig. 7I). Lung metastasis models and HE staining results indicated that the sh-circGDI2 + LGK-974 group had significantly fewer lung metastatic nodules compared to the other three groups (Fig. 7F, J). Liver orthotopic-implantation models results indicated that the sh-circGDI2 + LGK-974 group had significantly fewer liver metastatic nodules compared to the other three groups (Supporting Fig. S11C-D). All treatments exhibited favorable safety profiles regarding cell toxicity assays, organ histology and blood parameters (Supporting Fig. S11E-I). In summary, LGK-974 can inhibit the growth and metastasis of HCC cells in vivo, and this antitumor effect could be enhanced by the knockdown of circGDI2. Therefore, the combination of LGK-947 and the knockdown of circGDI2 emerges as a promising therapeutic approach to inhibit the growth and metastasis of HCC. This strategy targets the Wnt pathway key molecule, PORCN, employing a multidimensional approach.

## Discussion

In recent years, significant progress has been made in the research and treatment of HCC. However, the cancer-related mortality rate for HCC remains high, primarily due to the limited opportunities for curative surgical resection and the high postoperative recurrence rate. These challenges are closely related to the rapid proliferation and invasive metastasis of the tumor. CircRNAs are characterized by stability, specificity, diversity, and high expression levels [28]. These unique features have prompted the research into exploring the functions of circRNAs in HCC progression and their potential as diagnostic and prognostic biomarkers, as well as therapeutic targets. For instance, circITGB6 is found to potently promote EMT process and tumor metastasis in various models in vitro and in vivo by enhancing the mRNA stability of an EMT-promoting gene [29]. CircDOCK1 [2–27] is highly regulated by the epithelial splicing regulator ESPR1 in EMT and contributes to the suppression of the migratory capacity of cancer cells [30]. In our present study, we identified a novel circRNA-circGDI2 that was upregulated in HCC tissues by analyzing the RNA-sequencing results. The expression level of circGDI2 is significantly associated with serum AFP levels, presence of MVI and macrovascular invasion, BCLC stage, and tumor differentiation. But circGDI2 expression is independent on HBV/DNA copies/mL. We speculate that circGDI2 could drive tumor aggressiveness independently of viral factors to affect the Wnt/β-catenin signaling pathway. In addition, since HBV DNA copies/mL only reflect the viral replication status at hospital admission, there is considerable variation in antiviral treatments received by patients prior to admission. Additionally, patients with high circGDI2 expression have poorer prognosis. This study included 123 tissue samples with complete follow-up data. Although this is a single-center, retrospective cohort, the sample size was sufficient to detect significant associations (*p* < 0.05) between circGDI2 expression and clinicopathological parameters as well as survival outcomes. Both univariate and multivariate Cox regression analyses to evaluate the independent prognostic value of circGDI2. As shown in Table S3, after adjusting for key known prognostic factors in HCC, circGDI2 expression remained an independent risk factor for both OS and RFS in the multivariate analysis. Gain- and loss-of-function experiments further indicated that circGDI2 positively regulated the proliferation and invasion of HCC cells. In vivo experiments also showed that knocking down circGDI2 inhibited the growth and metastasis of HCC. Taken together, our results demonstrated that circGDI2 contributed to the progression and metastasis of HCC and can be considered as a prognostic factor for HCC patients.

The classic Wnt/β-catenin signaling pathway is highly conserved and plays a crucial role in maintaining homeostasis and regulating tissue development [31, 32]. Dysregulation of the Wnt pathway can promote the development of various liver diseases, including cancer progression. Most tumor cells exhibit genetic aberrations and enhanced Wnt pathway activity [33]. Cancer stem cells (CSCs), characterized by their self-renewal capability, are a common feature of all cancers. The Wnt signaling pathway plays a pivotal role in CSC proliferation, easily identifying those CSCs responsible for promoting tumor growth [34]. Tumor cell proliferation is promoted from both positive and negative aspects: the activating components that favor tumor growth in the Wnt pathway are stimulated, while most inhibitory components are found to have mutations [27, 35]. β-catenin is a key transcriptional coactivator in the classic Wnt signaling pathway, tightly regulating cell-cell adhesion [33]. It has an armadillo repeat domain that binds to E-cadherin, forming adhesive junctions between cells [36]. The C-terminal and N-terminal regions of the repeat domain are essential for β-catenin’s function. the C-terminal promotes β-catenin-mediated transcription by forming various binding factor complexes, while N-terminal phosphorylation facilitates β-catenin degradation. Notably, the gene encoding β-catenin, CTNNB1, is recognized as one of the most frequently mutated genes in HCC. β-catenin activation has been reported in 30% of HCC cases [37]. Our study further elucidates the relationship between the Wnt pathway and HCC.

PORCN is a member of the membrane-bound O-acyltransferase superfamily, capable of palmitoylating Wnt ligands, thus activating the Wnt pathway [38]. Inhibiting PORCN activity reduces the secretion of Wnt ligands, but does not affect the secretion of other classes of ligands [24]. Therefore, PORCN is considered a highly specific target for Wnt-driven cancers. In the canonical pathway, Wnt ligands bind to their receptors to inhibit the β-catenin destruction complex, ultimately leading to β-catenin driving the transcription of Wnt target genes that can trigger the occurrence of EMT. In our study, after knocking down circGDI2, we found that PORCN expression was significantly decreased, Wnt signaling became inactive, and at the molecular level, EMT-related molecules were negatively regulated. At the cellular level, HCC-LM9 and SK-Hep-1 cells became skeletal and less vigorous. Reducing PORCN expression with siRNA or inhibiting PORCN activity with LGK-974 can abolish the proliferative and metastatic effects of circGDI2 in HCC cells. Overall, our study suggested that circGDI2 acted as an important regulator of PORCN, thereby modulating the Wnt/β-catenin pathway in HCC.

CircRNAs act in many aspects of the gene expression flow from modulating transcription in the nucleus to translation in the cytoplasm. They function as sponges for miRNAs or proteins, platforms or scaffolds for proteins, regulators of transcription or translation [28]. The competing endogenous RNA hypothesis is the most well-studied mechanism. Our previous research demonstrated that circSETD3 acted as a sponge for microRNA-421, inhibiting HCC by activating the tumor suppressor signaling pathway MAPK14 [39]. Another study demonstrated that cGGNBP2 could encode the protein cGGNBP2-184aa, forming a positive feedback loop to facilitate intrahepatic cholangiocarcinoma progression [40]. However, circRNA do not have as many miRNA-binding sites as expected, and the level of target sites required for competing with miRNAs is relatively low, making their function as miRNA sponges highly controversial [41, 42]. In our study, although circGDI2 is highly expressed in HBV-related HCC tissues and cell lines, experimental results indicated no interaction between circGDI2 and AGO2, and circGDI2 had a low coding potential. Therefore, it cannot function as a miRNA sponge or encode protein. The interaction of circRNAs with protein is attracting increasing attention in various cancers [43–46]. Given that we identified HNRNPC as a binding protein of circGDI2 through RNA pull-down, MS, and RIP analysis. To map the domain mediating the interaction between circGDI2 and HNRNPC. Based on the secondary structure, we constructed truncations and confirmed that the nucleotide fragment 1-87aa of HNRNPC mediated the binding to the circGDI2.

Heterogeneous nuclear ribonucleoproteins (hnRNPs) are collectively referred to as a family of RBPs that play crucial roles in nucleic acid metabolism. They are essential for the regulation of gene expression and function in an additive manner, thus being considered as key molecules in tumor development [47]. HNRNPL enhances the stability of circMGA and CCL5 mRNA to regulate CD8 + T cells infiltration and immunotherapy in bladder cancer [48]. Interacting with HNRNPC, circTET2 was involved in the regulation of FAO and the mTORC1 signaling pathway to provide energy demands and promote the proliferation of CLL cells [43]. Our study firstly found that circGDI2 stabilized PORCN mRNA by binding to HNRNPC, thereby prolonging the half-life of PORCN mRNA and enhancing Wnt/β-catenin signaling to promote HCC proliferation and metastasis. Interestingly, overexpression of circGDI2 or knockdown of HNRNPC did not directly affect each other’s expression levels. However, knocking down HNRNPC made PORCN mRNA unstable, and overexpressing circGDI2 could rescue for this effect, suggesting that the up-regulation of PORCN by circGDI2 was dependent on HNRNPC and that circGDI2 played a vital role in synergizing with HNRNPC to regulate PORCN expression. Collectively, we demonstrated that circGDI2-HNRNPC complex enhanced the expression of PORCN. However, the topological structure of the circGDI2/HNRNPC complex requires further investigation, which may reveal whether circGDI2 plays a significant role in the conformational changes of HNRNPC.

The growth and invasion of HCC, as well as postoperative metastasis and recurrence, remain major factors affecting the survival of HCC patients [49, 50]. To establish effective methods to block the growth and metastasis of HCC, it is crucial to explore the molecular mechanisms underlying the biological functions associated with the malignant progression of HCC. Inhibition of the Wnt signaling pathway is an attractive therapeutic approach given its frequent deregulation in a wide range of tumor types [51]. Inhibitors have been developed to target different levels of the Wnt pathway [52]. Inhibition of PORCN prevents palmitoylation of Wnt ligands which in turn blocks the transport of Wnt to the extracellular membrane, thus prevents the immoderate production of β-catenin which contributes to control the aberrant cell growth [27]. PORCN inhibitors have been studied for the treatment of various solid tumors. However, to date, no drugs inhibiting PORCN have been applied in clinical settings. Only four molecules, LGK-974, ETC159, CGX1321, and RXC004, have entered the clinical trial phase [53–56]. Hayashi et al. reported on the impact of the LGK-974 on the metastasis of Ewing sarcoma. They found that LGK-974 at a concentration of 1 µM did not affect the in vitro proliferation of ES cells. However, it inhibited the expression of many genes considered to drive EMT and participate in metastasis [57]. Our results indicated that LGK-974 could effectively and safely inhibit the proliferation and metastasis of HCC cells. Moreover, previous studies provided a notion that simultaneous inhibition of multiple oncogenic pathways may lead to stronger pathway blockade [51, 58]. Our study provided more supports to the notion. On the basis of LGK-974, knocking down circGDI2 to reduce its binding with HNRNPC ultimately led to decreased stability of PORCN mRNA, more effectively inhibiting the Wnt/β-catenin signaling pathway. In other words, knocking down circGDI2 can enhance the sensitivity of HCC cells to LGK-974, which also explains the possible reason for the tumor’s resistance to LGK-974.

Our results showed that knocking down circGDI2 enhanced the anti-tumor efficiency of LGK-974 both in vitro and in vivo, indicating that circGDI2 may be a potential target for HCC. However, this study has several limitations. First, all clinical samples were collected from a single center and consisted exclusively of HBV-related HCC specimens, which may introduce potential biases affecting clinical outcomes. It needs for larger sample sizes to determine the relationship between circGDI2 expression levels and therapeutic response. We hope that future validation be conducted in multicenter, prospective cohorts. Second, this study identifies circGDI2 as a prognostic factor independent of conventional serological markers like AFP. However, its association with emerging molecular subtypes or specific driver gene mutations remains unknown and warrants future investigation. Third, although the in vitro and in vivo data from this study demonstrate encouraging synergistic efficacy and preliminary safety profiles, significant hurdles remain for clinical translation. Future studies will need to optimize the delivery systems for circGDI2-targeting agents (e.g., nanocarriers, liposomes) and systematically evaluate the pharmacokinetics, maximum tolerated dose, and potential organ toxicity of the combination regimen in more clinically relevant models, such as patient-derived xenograft (PDX) or humanized mouse models.

## Conclusion

**Fig. 8:**
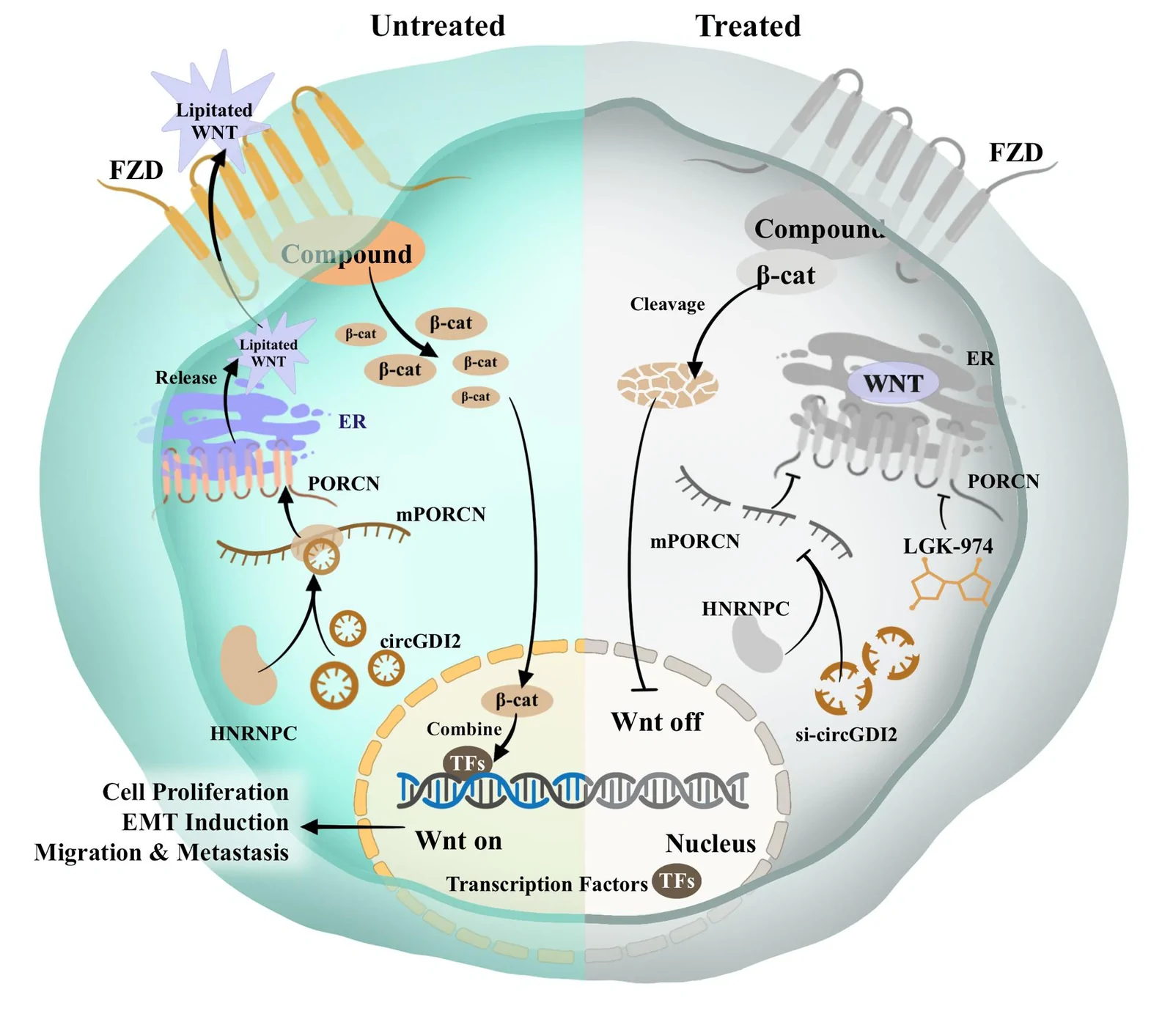
Summary figure of the mechanism of circGDI2 facilitates tumor progression of HCC. CircGDI2 interacts with HNRNPC to stabilize the PORCN mRNA, leading to the upregulation of PORCN, which further promote the Wnt/β-catenin pathway to induce HCC proliferation and metastasis. In this regard, knocking-down of circGDI2 combines with PORCN inhibitor (LGK-974) greatly enhance the anti-tumor effects by hindering the Wnt/β-catenin pathway. *ER* Endoplasmic reticulum, *EMT* Epithelial-Mesenchymal Transition

In summary, our study reveals that circGDI2 plays a tumor promoter in HCC development by interacting with HNRNPC to stabilize the PORCN mRNA, leading to the upregulation of PORCN, which further promotes the Wnt/β-catenin pathway in HCC (Fig. 8). Therefore, circGDI2 may serve as a diagnostic and prognostic biomarker for HCC patients. Moreover, the circGDI2-HNRNPC-PORCN-Wnt/β-catenin axis is a promising translational treatment target for further investigation in HCC.

## Materials and methods

### HBV-related HCC samples

This study utilized a total of 123 HBV-related HCC specimens and 55 corresponding adjacent normal tissues. All the HCC and adjacent normal tissue samples involved in this study were obtained from the biobank of West China Hospital, Sichuan University. These specimens were collected from patients who underwent curative HCC resection in the Department of Liver Surgery at West China Hospital from 2013 to 2016. The study was approved by the Biomedical Ethics Committee of West China Hospital (Ethic approval ID: 2022(1685)). Written informed consent was obtained from the patients or their relatives.

### Cell culture

HCC cell lines cells were maintained in Dulbecco’s modified Eagle medium/high-glucose medium (HyClone, Logan, UT, USA) supplemented with 10% fetal bovine serum (FBS) (PAN-Biotek, Aidenbach, Bavaria) and antibiotics (1% penicillin/streptomycin; HyClone) in a humidified incubator with 5% CO2 at 37 °C.

### Cell transfection

Small interfering RNAs (siRNAs) targeting circGDI2, HNRNPC and PORCN were designed and synthesised by RiboBio (Guangzhou, China). The siRNA sequences are listed in Table S9. siRNAs were transfected using GeneMute (SignaGen Laboratories, Rockville, MD, USA), according to the manufacturer’s instructions. The lentiviruses used to knockdown circGDI2 and overexpress circGDI2 and PORCN were constructed by GeneChem (Shanghai, China) and infected following the manufacturer’s protocol.

### Quantitative real-time (qRT)-PCR

Genomic DNA (gDNA) was isolated using the PureLink™ Genomic DNA Mini Kit (Thermo Fisher Scientific, Waltham, MA, USA), according to the manufacturer’s instructions. Total RNA was extracted using TRIzol (Invitrogen Life Technologies Inc., Germany). Reverse transcription was performed using HiScript III RT SuperMix for qPCR (+ gDNA wiper) Kit (Vazyme Biotech Co. Ltd., Nanjing, China). Nuclear and cytoplasmic fractions were isolated using the PARIS™ Kit (Thermo Fisher Scientific). qRT-PCR was performed in triplicate using 2× ChamQ Universal SYBR qPCR Master Mix (Vazyme Biotech Co. Ltd.) and the CFX Connect Real-Time System (Bio-Rad, Hercules, CA, USA). The primers used in this study are listed in Table S10.

### Actinomycin D assay

HCC cells were cultured in 6-well plate at a density of 2 × 10^5^ cells/well overnight and treated with 2 µg/ml actinomycin D (MCE, USA) for 4, 8, 12, 16 and 24 h. The expression of circGDI2 and GDI2 mRNA was analyzed by qRT-PCR.

### RNase R treatment

The extracted RNA from HCC cells was divided into two groups. In the RNase R group, 3 µg of RNA was treated with 10 U RNase R (20 U/µL; Epicenter, Madison, WI, USA), at 37 °C for 45 min, followed by 70 °C for 10 min to deactivate RNase R. Then, qRT-PCR was conducted to analyze the expression of circGDI2 and GDI2 mRNA.

### RNA cytosolic/nuclear fraction assay

The RNA cytosolic/nuclear fraction assay was performed using the PARIS™ Kit (Thermo Fisher Scientific, Waltham, MA, USA), following the manufacturer’s instructions. Subsequently, RNA derived from the cytoplasmic cell fraction and nuclear pellet was purified using a filter cartridge system and subjected to qRT-PCR analysis.

### Western blotting

Tissue or cell samples were lysed by RIPA Lysis Buffer (Beyotime, China) with PMSF. We performed Western blot according to the manufacturer’s protocol and our previous study [59]. The antibodies are listed in Table S11.

### Immunohistochemistry (IHC)

The Xenograft tissues were first deparaffinized and rehydrated with alcohol before antigen retrieval using citrate antigen retrieval solution (Beyotime, China). Subsequently, the sections were blocked with 5% normal goat serum containing 0.1% Triton X-100 and 3% H2O2 in PBS at room temperature for 1 h. The sections were then incubated overnight at 4℃ with specific primary antibodies, followed by incubation with secondary antibodies. Finally, immunohistochemical staining was examined using an optical microscope.

### Fluorescent in situ hybridization (FISH)

Cells were washed with PBS and fixed with 4% paraformaldehyde at room temperature for 20 min. The cells were then permeabilized with PBS containing 0.2% Triton X-100 on ice for 10 min. Fluorescence in situ hybridization (FISH) was performed using a kit (RiboBio, Guangzhou, China) according to the manufacturer’s instructions. An anti-circGDI2 oligodeoxynucleotide probe was synthesized with Cy3 as the conjugate. Cy3-labeled anti-18 S or U6 oligonucleotide probes (RiboBio) were used as controls. The cells were then hybridized in a humidified chamber at 50 °C for 16 h using the hybridization buffer. Subsequently, the cells were washed at 50 °C in 25% deionized formamide/2×SSC for 30 min, followed by washing in 2×SSC at 42 °C for 30 min. For microscopy, the cells on the coverslip were stained with DAPI and scanned using a confocal laser scanning microscope (Nikon, Tokyo, Japan).

### Immunofluorescence (IF) staining

Cells were seeded onto coverslips placed in a 24-well plate, and incubated for 24 h. Then the cells were fixed with 4% paraformaldehyde for 30 min, permeabilized with 0.2% Triton X-100 for 10 min, and blocked with 3% bovine serum albumin for 1 h. Cells were incubated with specific antibodies overnight at 4 °C, followed by incubation with fluorescence-conjugated secondary antibodies at 37 °C for 1 h. Nuclei were stained with 4,6-diamidino-2-phenylindole (DAPI) for 10 min. After sealing, images were obtained using a confocal laser scanning microscope (Nikon).

### CCK-8 assay

Cells were seeded at a density of 2 × 10^3^ cells/well in a 96-well plate the day before. Subsequently, 100 µl medium containing 10 µl CCK8 reagent (Oriscience, China) was added into each well daily. After 1.5 h incubation, the absorbance at 450 nm was measured by the Eon™ Microplate Reader (BioTek, Whiting, VT, USA).

### Edu assay

EdU cell proliferation kit (RiboBio, China) was used for EdU assay. Cells were planted in 24-well plates at 80% confluence, cultured for 24 h, and then treated with 10µM EdU in medium for 2 h. The cells were fixed in 4% paraformaldehyde and permeabilized with 0.3% Triton in PBS for 15 min. Sequentially the cells were stained with Alexa Fluor 555 azide for 30 min and DAPI for 10 min in the dark. The images were captured under a fluorescence microscope.

### Clone formation assay

Cells were seeded in 6-well plates at a density of 1 × 10^3^/well and incubated for 10–14 days according to cell type. The cells were fixed and then stained with Crystal Violet Staining Solution (Beyotime, China) for 30 min. The colony number was counted by Image J (National Institutes of Health, Bethesda, MD, USA) software.

### Transwell migration and Matrigel invasion assays

For Transwell migration assay, 3 × 10^4^ HCC cells were suspended in 300 µL of serum-free medium and seeded into the upper chamber (pore size, 8 μm) (Millipore, Billerica, MA, USA). The bottom chamber contained 600 µL of medium containing 10% FBS as a chemoattractant. After 24 h, cells on the lower surface of the upper chamber were fixed with 4% paraformaldehyde, stained with 0.05% Crystal Violet Staining Solution, and imaged at 100× magnification. At least three random fields were photographed. Migrated cell numbers were counted using Image J. A similar protocol was performed for Matrigel invasion assay, except 30 µL of diluted Matrigel (BD Bioscience, Bedford, MA, USA) was added to the upper chamber before cell seeding.

### Wound healing assay

Confluent monolayer cells in 6-well plates were wounded using a 200 µL pipette tip. After washing with PBS twice, cells were cultured in medium containing 3% FBS. Images were acquired using an inverted microscope (Carl Zeiss, Jena, Germany) at 0, 24 and 48 h after wounding. At least three separate fields were photographed. The relative healed area was calculated using ImageJ and normalized to 0 h control.

### RNA pull-down assay

The biotin-labeled circGDI2 probe (GATACATTCCTTGCAAATCC-/3bio/) and control probe (RiboBio, China) were incubated with streptavidin magnetic beads (RiboBio, China) at room temperature for 30 min to generate probe-coated beads. Lysates from HCC cells were saved as a 100ul input group, and the rest of the lysates were incubated with probe-coated beads at 4 °C overnight. Then, the pulled down RNA was extracted and analyzed by qRT–PCR. The pulled down proteins underwent analysis through silver staining, mass spectrometry and western blotting.

### RNA immunoprecipitation (RIP) assay

RIP assay was performed using the Magna RIP™ RNA-Binding Protein Immunoprecipitation Kit (Millipore) following the manufacturer’s instructions. Briefly, magnetic beads were sequentially incubated with IgG and HNRNPC antibody and prepared cell lysates. Enriched RNA was isolated using TRIzol reagent and quantified by qRT-PCR. Immunoglobulin G antibody served as the negative control.

### Animal models

Male BALB/c nude mice (5–6 weeks old) were purchased from HFK Bioscience (Beijing, China) and maintained under specific pathogen-free conditions. All animal experiments were approved by the Animal Care Committee of Sichuan University. To establish xenograft models, 5 × 10^6^ stably transfected cells were injected subcutaneously into the right scapular region of nude mice, and samples were collected approximately 4 weeks later for pathological examination. To establish liver orthotopic-implantation models, 1.5 × 10^6^ stably transfected cells were injected into the liver of nude mice. Four weeks later, liver lobes containing tumors were harvested and subjected to H&E staining. To establish lung metastasis models, 2 × 10^6^ stably transfected cells HCC cells were injected into the tail vein of nude mice. Eight weeks later, the mice were sacrificed. Resected lung specimens were stained with H&E staining.

### Statistical analysis

Continuous variables were expressed as mean ± SD and compared using Student’s t-test. Categorical variables were expressed as numbers and percentages and compared using the chi-square test or Fisher’s exact test, as appropriate. Correlations were determined using Pearson’s correlation coefficients. The optimal cut-off value for circGDI2 expression in HBV-related HCC tissues was determined using X-tile software (version 3.6.1) (Yale University, New Haven, CT, USA). Survival curves were plotted using the Kaplan–Meier method and compared using the log-rank test. All statistical tests were two-tailed. A p-value < 0.05 was considered statistically significant. All analyses were performed on SPSS (version 21.0) (IBM Corp., Armonk, NY, USA) or GraphPad Prism (version 8.0) (GraphPad Software, La Jolla, CA, USA).

## Supplementary Information

Supplementary Material 1.

Supplementary Material 2.

Supplementary Material 3.

Supplementary Material 4.

Supplementary Material 5.

Supplementary Material 6.

Supplementary Material 7.

## Data Availability

The datasets generated and analyzed during the current study are available from the corresponding author on reasonable request.

## Abbreviations

AFP: A-fetoprotein
CircRNA: Circular RNA
CSC: Cancer stem cell
EEMT: epithelial-mesenchymal transition
FISH: Fluorescence in situ hybridization
HCC: Hepatocellular carcinoma
HNRNPC: Heterogeneous nuclear ribonucleoprotein C
hnRNP: Heterogeneous nuclear ribonucleoproteins
IF: Immunofluorescence
lncRNA: Ong non-coding RNA
IHC: Immunohistochemistry
miRNA: MicroRNA
MS: Mass spectrometry
MVI: Microvascular invasion
ncRNA: Non-coding RNA
OS: Overall survival
PORCN: Porcupine O-acyltransferase
RFS: Recurrence-free survival
qRT-PCR: Quantitative real-time polymerase chain reaction
RBP: RNA binding proteins
RIP: RNA immunoprecipitation
SiRNA: Small interfering RNA
Wt: Wild type

## References

[1] Bray F, Ferlay J, Soerjomataram I, Siegel RL, Torre LA, Jemal A. Global cancer statistics 2018: GLOBOCAN estimates of incidence and mortality worldwide for 36 cancers in 185 countries. CA Cancer J Clin. 2018;68:394–424. 30207593 10.3322/caac.21492

[2] Llovet JM, Zucman-Rossi J, Pikarsky E, Sangro B, Schwartz M, Sherman M, Gores G. Hepatocellular carcinoma. Nat Rev Dis Primers. 2016;2:16018. 27158749 10.1038/nrdp.2016.18

[3] Franssen B, Alshebeeb K, Tabrizian P, Marti J, Pierobon ES, Lubezky N, Roayaie S, et al. Differences in surgical outcomes between hepatitis B- and hepatitis C-related hepatocellular carcinoma: a retrospective analysis of a single North American center. Ann Surg. 2014;260:650–6. discussion 656–658. 25203882 10.1097/SLA.0000000000000917

[4] Adelman K, Egan E. Non-coding RNA: More uses for genomic junk. Nature. 2017;543:183–5. 28277509 10.1038/543183a

[5] Shang Q, Yang Z, Jia R, Ge S. The novel roles of circRNAs in human cancer. Mol Cancer. 2019;18:6. 30626395 10.1186/s12943-018-0934-6 PMC6325800

[6] Hansen TB, Jensen TI, Clausen BH, Bramsen JB, Finsen B, Damgaard CK, Kjems J. Natural RNA circles function as efficient microRNA sponges. Nature. 2013;495:384–8. 23446346 10.1038/nature11993

[7] Yang Y, Gao X, Zhang M, Yan S, Sun C, Xiao F, Huang N, et al. Novel Role of FBXW7 Circular RNA in Repressing Glioma Tumorigenesis. J Natl Cancer Inst. 2018;110:304–15. 28903484 10.1093/jnci/djx166 PMC6019044

[8] Liu S, Wang Y, Wang T, Shi K, Fan S, Li C, Chen R, et al. CircPCNXL2 promotes tumor growth and metastasis by interacting with STRAP to regulate ERK signaling in intrahepatic cholangiocarcinoma. Mol Cancer. 2024;23:35. 38365721 10.1186/s12943-024-01950-y PMC10873941

[9] Xu J, Ji L, Liang Y, Wan Z, Zheng W, Song X, Gorshkov K, et al. CircRNA-SORE mediates sorafenib resistance in hepatocellular carcinoma by stabilizing YBX1. Signal Transduct Target Ther. 2020;5:298. 33361760 10.1038/s41392-020-00375-5 PMC7762756

[10] Lei K, Liang R, Liang J, Lu N, Huang J, Xu K, Tan B, et al. CircPDE5A-encoded novel regulator of the PI3K/AKT pathway inhibits esophageal squamous cell carcinoma progression by promoting USP14-mediated de-ubiquitination of PIK3IP1. J Exp Clin Cancer Res. 2024;43:124. 38658954 10.1186/s13046-024-03054-3 PMC11040784

[11] Chen J, Li Y, Zheng Q, Bao C, He J, Chen B, Lyu D, et al. Circular RNA profile identifies circPVT1 as a proliferative factor and prognostic marker in gastric cancer. Cancer Lett. 2017;388:208–19. 27986464 10.1016/j.canlet.2016.12.006

[12] Hsiao KY, Lin YC, Gupta SK, Chang N, Yen L, Sun HS, Tsai SJ. Noncoding Effects of Circular RNA CCDC66 Promote Colon Cancer Growth and Metastasis. Cancer Res. 2017;77:2339–50. 28249903 10.1158/0008-5472.CAN-16-1883 PMC5910173

[13] Li J, Wang X, Shi L, Liu B, Sheng Z, Chang S, Cai X, et al. A Mammalian Conserved Circular RNA CircLARP1B Regulates Hepatocellular Carcinoma Metastasis and Lipid Metabolism. Adv Sci (Weinh). 2024;11:e2305902. 37953462 10.1002/advs.202305902 PMC10787103

[14] Zhao Z, Yang W, Kong R, Zhang Y, Li L, Song Z, Chen H, et al. circEIF3I facilitates the recruitment of SMAD3 to early endosomes to promote TGF-β signalling pathway-mediated activation of MMPs in pancreatic cancer. Mol Cancer. 2023;22:152. 37689715 10.1186/s12943-023-01847-2 PMC10492306

[15] Hu X, Chen G, Huang Y, Cheng Q, Zhuo J, Su R, He C, et al. Integrated Multiomics Reveals Silencing of has_circ_0006646 Promotes TRIM21-Mediated NCL Ubiquitination to Inhibit Hepatocellular Carcinoma Metastasis. Adv Sci (Weinh). 2024;11:e2306915. 38357830 10.1002/advs.202306915 PMC11040345

[16] Li H, Xu L, Yi P, Li L, Yan T, Xie L, Zhu Z. High-throughput circular RNA sequencing reveals the profiles of circular RNA in non-cirrhotic hepatocellular carcinoma. BMC Cancer. 2022;22:857. 35931993 10.1186/s12885-022-09909-2 PMC9356431

[17] Wang C, Liu WR, Tan S, Zhou JK, Xu X, Ming Y, Cheng J, et al. Characterization of distinct circular RNA signatures in solid tumors. Mol Cancer. 2022;21:63. 35236349 10.1186/s12943-022-01546-4 PMC8889743

[18] Conn SJ, Pillman KA, Toubia J, Conn VM, Salmanidis M, Phillips CA, Roslan S, et al. The RNA binding protein quaking regulates formation of circRNAs. Cell. 2015;160:1125–34. 25768908 10.1016/j.cell.2015.02.014

[19] Ashwal-Fluss R, Meyer M, Pamudurti NR, Ivanov A, Bartok O, Hanan M, Evantal N, et al. circRNA biogenesis competes with pre-mRNA splicing. Mol Cell. 2014;56:55–66. 25242144 10.1016/j.molcel.2014.08.019

[20] MargvelaniG, Maquera KAA, Welden JR, Rodgers DW, Stamm S. Translation of circular RNAs. Nucleic Acids Res 2025;53. 10.1093/nar/gkae1167. 10.1093/nar/gkae1167 PMC11724312 39660652

[21] Zheng X, Huang M, Xing L, Yang R, Wang X, Jiang R, Zhang L, et al. The circRNA circSEPT9 mediated by E2F1 and EIF4A3 facilitates the carcinogenesis and development of triple-negative breast cancer. Mol Cancer. 2020;19:73. 32264877 10.1186/s12943-020-01183-9 PMC7137343

[22] Wei W, Liu K, Huang X, Tian S, Wang H, Zhang C, Ye J, et al. EIF4A3-mediated biogenesis of circSTX6 promotes bladder cancer metastasis and cisplatin resistance. J Exp Clin Cancer Res. 2024;43:2. 38163881 10.1186/s13046-023-02932-6 PMC10759346

[23] Jiang X, Guo S, Wang S, Zhang Y, Chen H, Wang Y, Liu R, et al. EIF4A3-Induced circARHGAP29 Promotes Aerobic Glycolysis in Docetaxel-Resistant Prostate Cancer through IGF2BP2/c-Myc/LDHA Signaling. Cancer Res. 2022;82:831–45. 34965937 10.1158/0008-5472.CAN-21-2988

[24] Madan B, Virshup DM. Targeting Wnts at the source–new mechanisms, new biomarkers, new drugs. Mol Cancer Ther. 2015;14:1087–94. 25901018 10.1158/1535-7163.MCT-14-1038

[25] Wang X, Xue X, Pang M, Yu L, Qian J, Li X, Tian M, et al. Epithelial-mesenchymal plasticity in cancer: signaling pathways and therapeutic targets. MedComm. 2020;2024(5):e659. 10.1002/mco2.659 PMC11292400 39092293

[26] Chen LL. The expanding regulatory mechanisms and cellular functions of circular RNAs. Nat Rev Mol Cell Biol. 2020;21:475–90. 32366901 10.1038/s41580-020-0243-y

[27] Shah K, Panchal S, Patel B. Porcupine inhibitors: Novel and emerging anti-cancer therapeutics targeting the Wnt signaling pathway. Pharmacol Res. 2021;167:105532. 33677106 10.1016/j.phrs.2021.105532

[28] Liu CX, Chen LL. Circular RNAs: Characterization, cellular roles, and applications. Cell. 2022;185:2016–34. 35584701 10.1016/j.cell.2022.04.021

[29] Li K, Guo J, Ming Y, Chen S, Zhang T, Ma H, Fu X, et al. A circular RNA activated by TGFβ promotes tumor metastasis through enhancing IGF2BP3-mediated PDPN mRNA stability. Nat Commun. 2023;14:6876. 37898647 10.1038/s41467-023-42571-1 PMC10613289

[30] Liu D, Dredge BK, Bert AG, Pillman KA, Toubia J, Guo W, Dyakov BJA, et al. ESRP1 controls biogenesis and function of a large abundant multiexon circRNA. Nucleic Acids Res. 2024;52:1387–403. 38015468 10.1093/nar/gkad1138 PMC10853802

[31] Liu D, Chen L, Zhao H, Vaziri ND, Ma SC, Zhao YY. Small molecules from natural products targeting the Wnt/β-catenin pathway as a therapeutic strategy. Biomed Pharmacother. 2019;117:108990. 31226638 10.1016/j.biopha.2019.108990

[32] Holzem M, Boutros M, Holstein TW. The origin and evolution of Wnt signalling. Nat Rev Genet. 2024;25:500–12. 38374446 10.1038/s41576-024-00699-w

[33] Thompson MD, Monga SP. WNT/beta-catenin signaling in liver health and disease. Hepatology. 2007;45:1298–305. 17464972 10.1002/hep.21651

[34] Chu X, Tian W, Ning J, Xiao G, Zhou Y, Wang Z, Zhai Z, et al. Cancer stem cells: advances in knowledge and implications for cancer therapy. Signal Transduct Target Ther. 2024;9:170. 38965243 10.1038/s41392-024-01851-y PMC11224386

[35] Polakis P. Drugging Wnt signalling in cancer. Embo j. 2012;31:2737–46. 22617421 10.1038/emboj.2012.126 PMC3380214

[36] Niessen CM, Gottardi CJ. Molecular components of the adherens junction. Biochim Biophys Acta. 2008;1778:562–71. 18206110 10.1016/j.bbamem.2007.12.015 PMC2276178

[37] Rialdi A, Duffy M, Scopton AP, Fonseca F, Zhao JN, Schwarz M, Molina-Sanchez P, et al. WNTinib is a multi-kinase inhibitor with specificity against β-catenin mutant hepatocellular carcinoma. Nat Cancer. 2023;4:1157–75. 37537299 10.1038/s43018-023-00609-9 PMC10948969

[38] Torres VI, Godoy JA, Inestrosa NC. Modulating Wnt signaling at the root: Porcupine and Wnt acylation. Pharmacol Ther. 2019;198:34–45. 30790642 10.1016/j.pharmthera.2019.02.009

[39] Xu L, Feng X, Hao X, Wang P, Zhang Y, Zheng X, Li L, et al. CircSETD3 (Hsa_circ_0000567) acts as a sponge for microRNA-421 inhibiting hepatocellular carcinoma growth. J Exp Clin Cancer Res. 2019;38:98. 30795787 10.1186/s13046-019-1041-2 PMC6385474

[40] Li H, Lan T, Liu H, Liu C, Dai J, Xu L, Cai Y, et al. IL-6-induced cGGNBP2 encodes a protein to promote cell growth and metastasis in intrahepatic cholangiocarcinoma. Hepatology. 2022;75:1402–19. 34758510 10.1002/hep.32232 PMC9306806

[41] Denzler R, Agarwal V, Stefano J, Bartel DP, Stoffel M. Assessing the ceRNA hypothesis with quantitative measurements of miRNA and target abundance. Mol Cell. 2014;54:766–76. 24793693 10.1016/j.molcel.2014.03.045 PMC4267251

[42] Li X, Yang L, Chen LL. The Biogenesis, Functions, and Challenges of Circular RNAs. Mol Cell. 2018;71:428–42. 30057200 10.1016/j.molcel.2018.06.034

[43] Wu Z, Zuo X, Zhang W, Li Y, Gui R, Leng J, Shen H, et al. m6A-Modified circTET2 Interacting with HNRNPC Regulates Fatty Acid Oxidation to Promote the Proliferation of Chronic Lymphocytic Leukemia. Adv Sci (Weinh). 2023;10:e2304895. 37821382 10.1002/advs.202304895 PMC10700176

[44] Yao X, Liu H, Wang Z, Lu F, Chen W, Feng Q, Miao Y, et al. Circular RNA EIF3I promotes papillary thyroid cancer progression by interacting with AUF1 to increase Cyclin D1 production. Oncogene. 2023;42:3206–18. 37697064 10.1038/s41388-023-02830-3

[45] Meng X, Xiao W, Sun J, Li W, Yuan H, Yu T, Zhang X, et al. CircPTK2/PABPC1/SETDB1 axis promotes EMT-mediated tumor metastasis and gemcitabine resistance in bladder cancer. Cancer Lett. 2023;554:216023. 36436682 10.1016/j.canlet.2022.216023

[46] Luo Y, Zhu Q, Xiang S, Wang Q, Li J, Chen X, Yan W, et al. Downregulated circPOKE promotes breast cancer metastasis through activation of the USP10-Snail axis. Oncogene. 2023;42:3236–51. 37717099 10.1038/s41388-023-02823-2

[47] Geuens T, Bouhy D, Timmerman V. The hnRNP family: insights into their role in health and disease. Hum Genet. 2016;135:851–67. 27215579 10.1007/s00439-016-1683-5 PMC4947485

[48] Sun J, Zhang H, Wei W, Xiao X, Huang C, Wang L, Zhong H, et al. Regulation of CD8(+) T cells infiltration and immunotherapy by circMGA/HNRNPL complex in bladder cancer. Oncogene. 2023;42:1247–62. 36869127 10.1038/s41388-023-02637-2

[49] Hlady RA, Robertson KD. A Three-Pronged Epigenetic Approach to the Treatment of Hepatocellular Carcinoma. Hepatology. 2018;68:1226–8. 30070376 10.1002/hep.30133

[50] Finn RS, Zhu AX, Farah W, Almasri J, Zaiem F, Prokop LJ, Murad MH, et al. Therapies for advanced stage hepatocellular carcinoma with macrovascular invasion or metastatic disease: A systematic review and meta-analysis. Hepatology. 2018;67:422–35. 28881497 10.1002/hep.29486

[51] Hao J, Han X, Huang H, Yu X, Fang J, Zhao J, Prayson RA, et al. Sema3C signaling is an alternative activator of the canonical WNT pathway in glioblastoma. Nat Commun. 2023;14:2262. 37080989 10.1038/s41467-023-37397-w PMC10119166

[52] Zhan T, Rindtorff N, Boutros M. Wnt signaling in cancer. Oncogene. 2017;36:1461–73. 27617575 10.1038/onc.2016.304 PMC5357762

[53] Harb J, Lin PJ, Hao J. Recent Development of Wnt Signaling Pathway Inhibitors for Cancer Therapeutics. Curr Oncol Rep. 2019;21:12. 30715618 10.1007/s11912-019-0763-9

[54] Tammela T, Sanchez-Rivera FJ, Cetinbas NM, Wu K, Joshi NS, Helenius K, Park Y, et al. A Wnt-producing niche drives proliferative potential and progression in lung adenocarcinoma. Nature. 2017;545:355–9. 28489818 10.1038/nature22334 PMC5903678

[55] Yang XG, Zhu LC, Wang YJ, Li YY, Wang D. Current Advance of Therapeutic Agents in Clinical Trials Potentially Targeting Tumor Plasticity. Front Oncol. 2019;9:887. 31552191 10.3389/fonc.2019.00887 PMC6746935

[56] GoldsberryWN, Londoño A, Randall TD, Norian LA, Arend RC. A Review of the Role of Wnt in Cancer Immunomodulation. Cancers (Basel) 2019;11. 10.3390/cancers11060771. 10.3390/cancers11060771 PMC6628296 31167446

[57] Hayashi M, Baker A, Goldstein SD, Albert CM, Jackson KW, McCarty G, Kahlert UD, et al. Inhibition of porcupine prolongs metastasis free survival in a mouse xenograft model of Ewing sarcoma. Oncotarget. 2017;8:78265–76. 29108227 10.18632/oncotarget.19432 PMC5667961

[58] Du J, Lan T, Liao H, Feng X, Chen X, Liao W, Hou G, et al. CircNFIB inhibits tumor growth and metastasis through suppressing MEK1/ERK signaling in intrahepatic cholangiocarcinoma. Mol Cancer. 2022;21:18. 35039066 10.1186/s12943-021-01482-9 PMC8762882

[59] Xu L, Wang P, Li L, Li L, Huang Y, Zhang Y, Zheng X, et al. circPSD3 is a promising inhibitor of uPA system to inhibit vascular invasion and metastasis in hepatocellular carcinoma. Mol Cancer. 2023;22:174. 37884951 10.1186/s12943-023-01882-z PMC10601121

