# Supplementary figures and images for: Targeting circGDI2 disrupt HNRNPC-mediated mPORCN stabilization and enhance LGK-974 anti-tumor therapy in hepatocellular carcinoma
Source: Mol Cancer. 2026 Mar 10;25:110. doi: 10.1186/s12943-026-02638-1 (PMC13088843; doi:10.1186/s12943-026-02638-1)

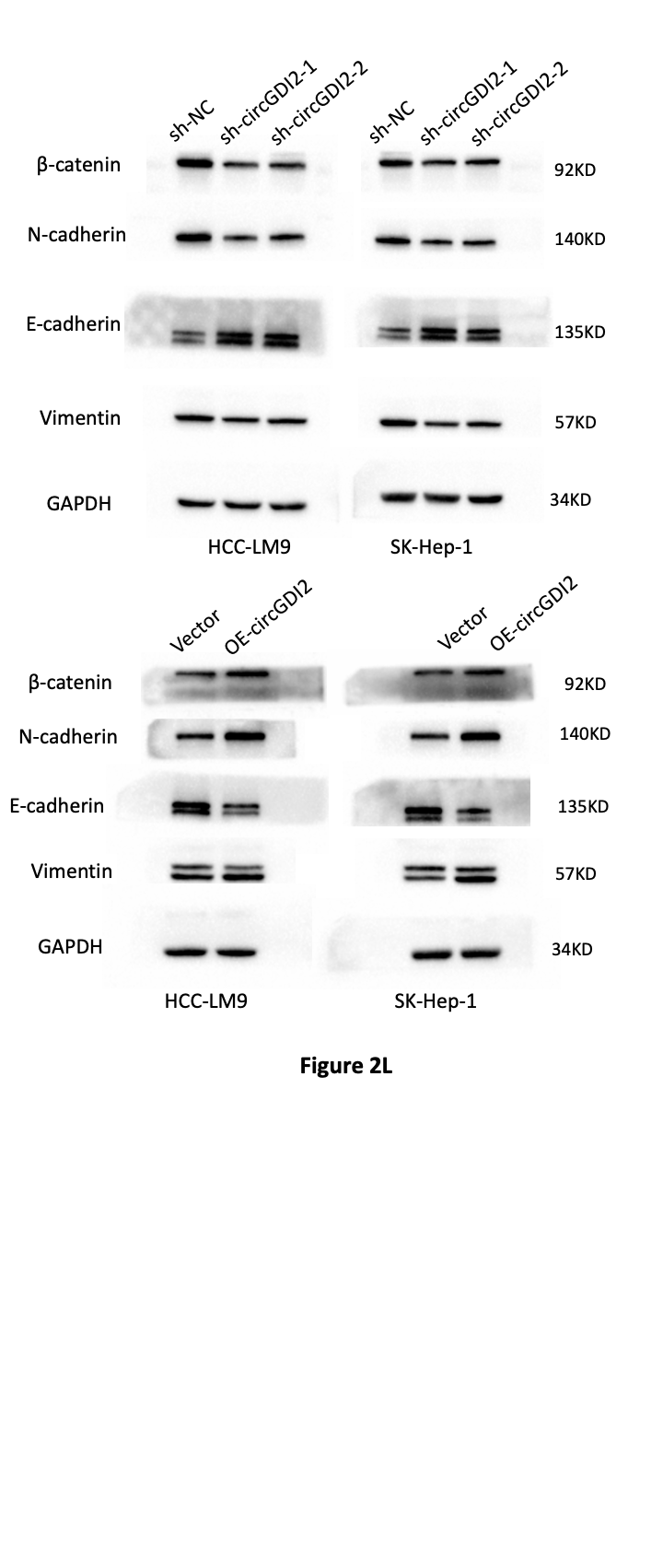


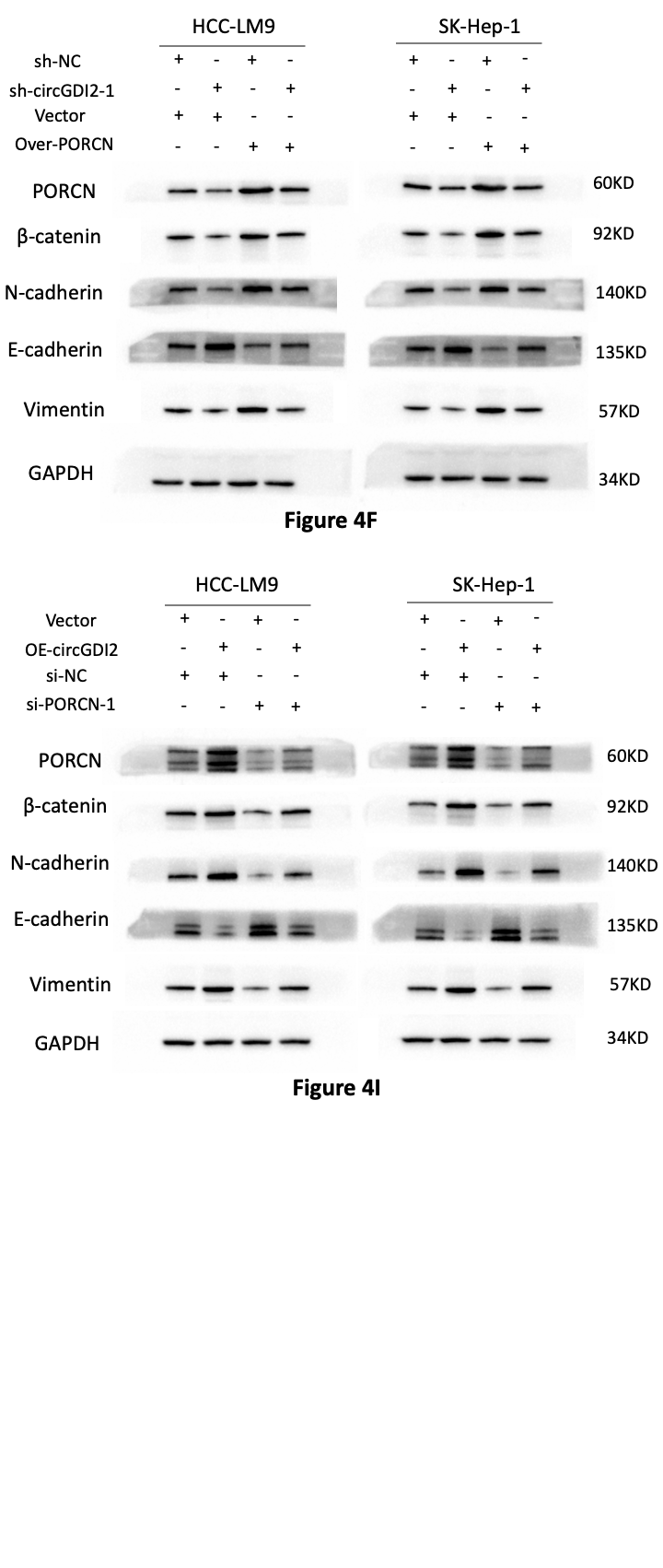


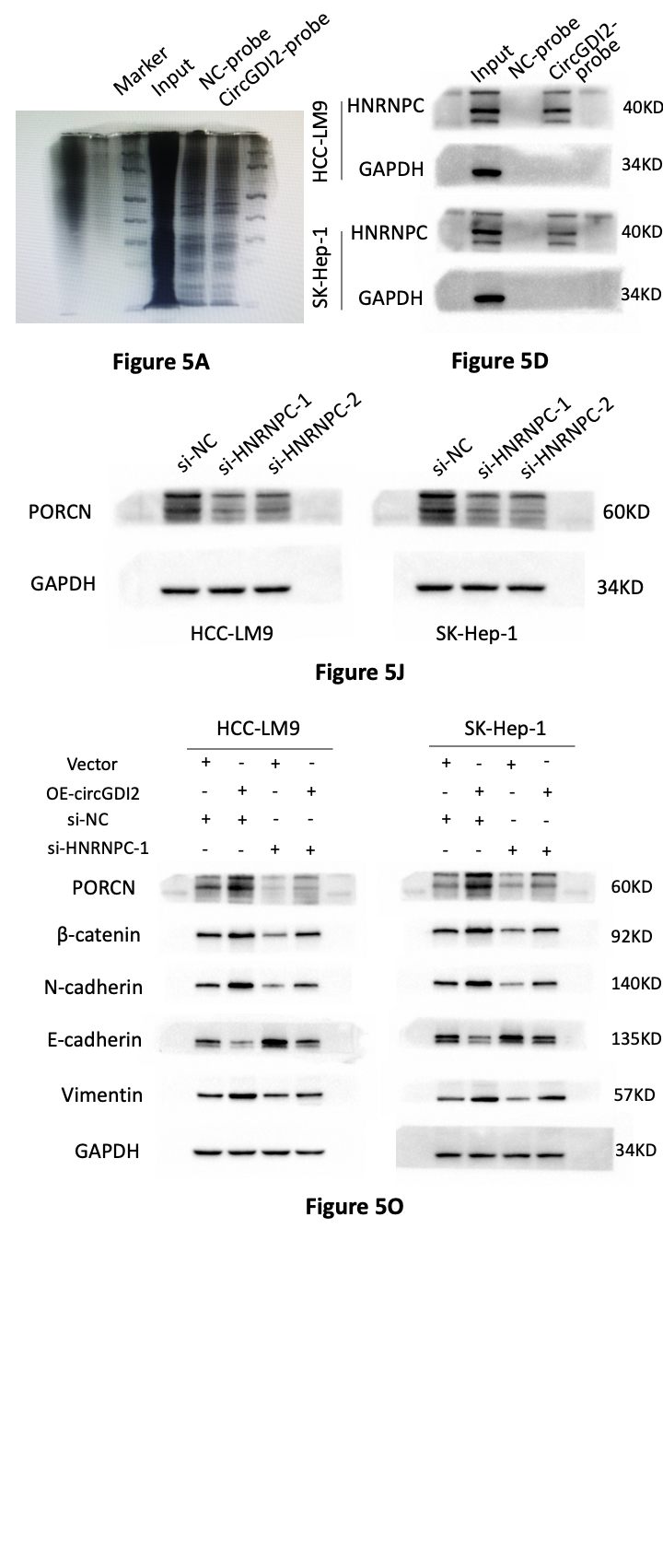


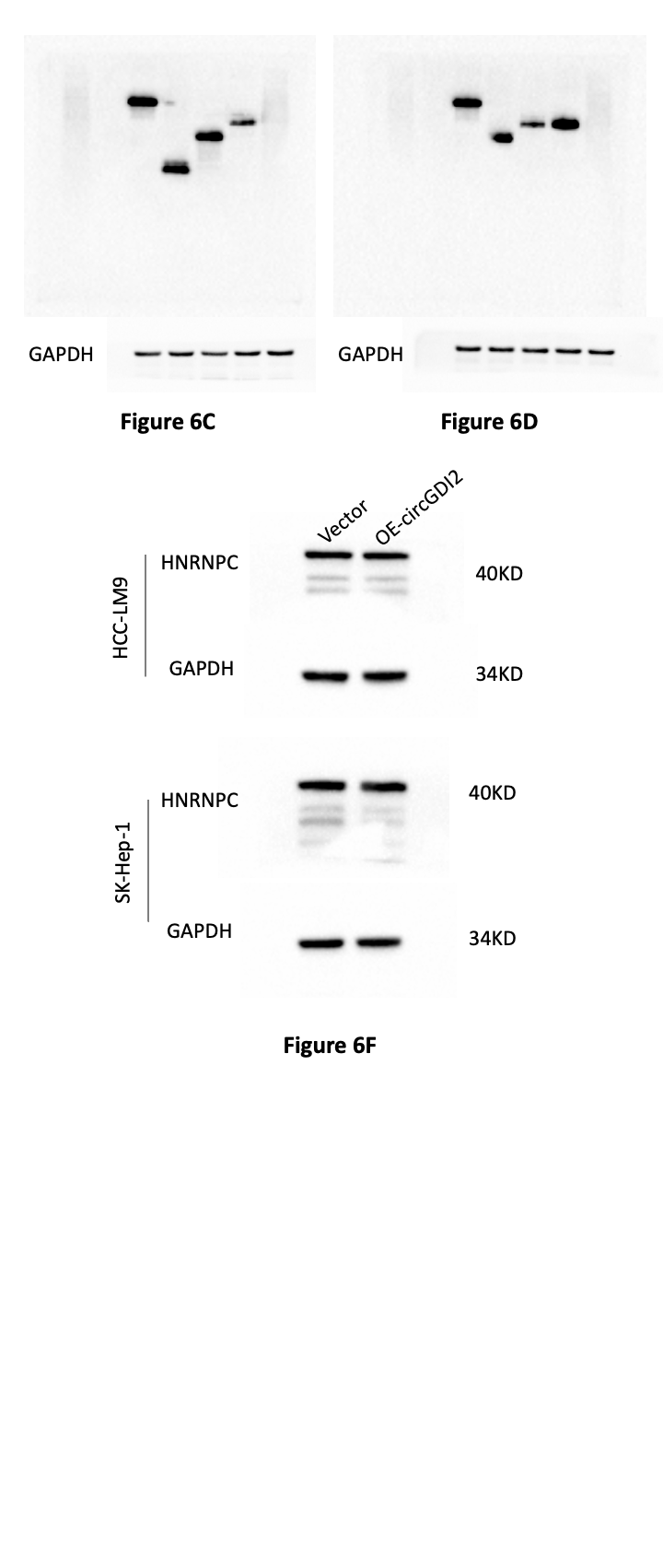


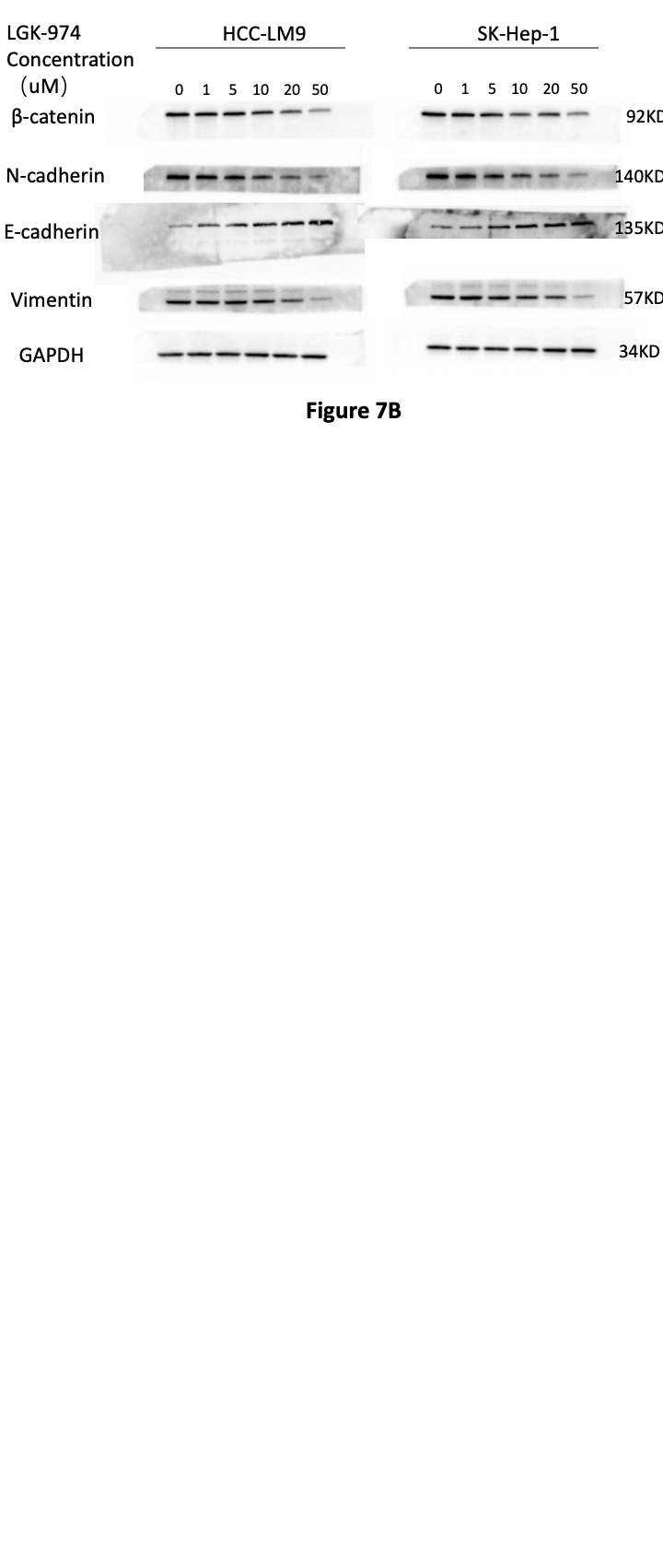


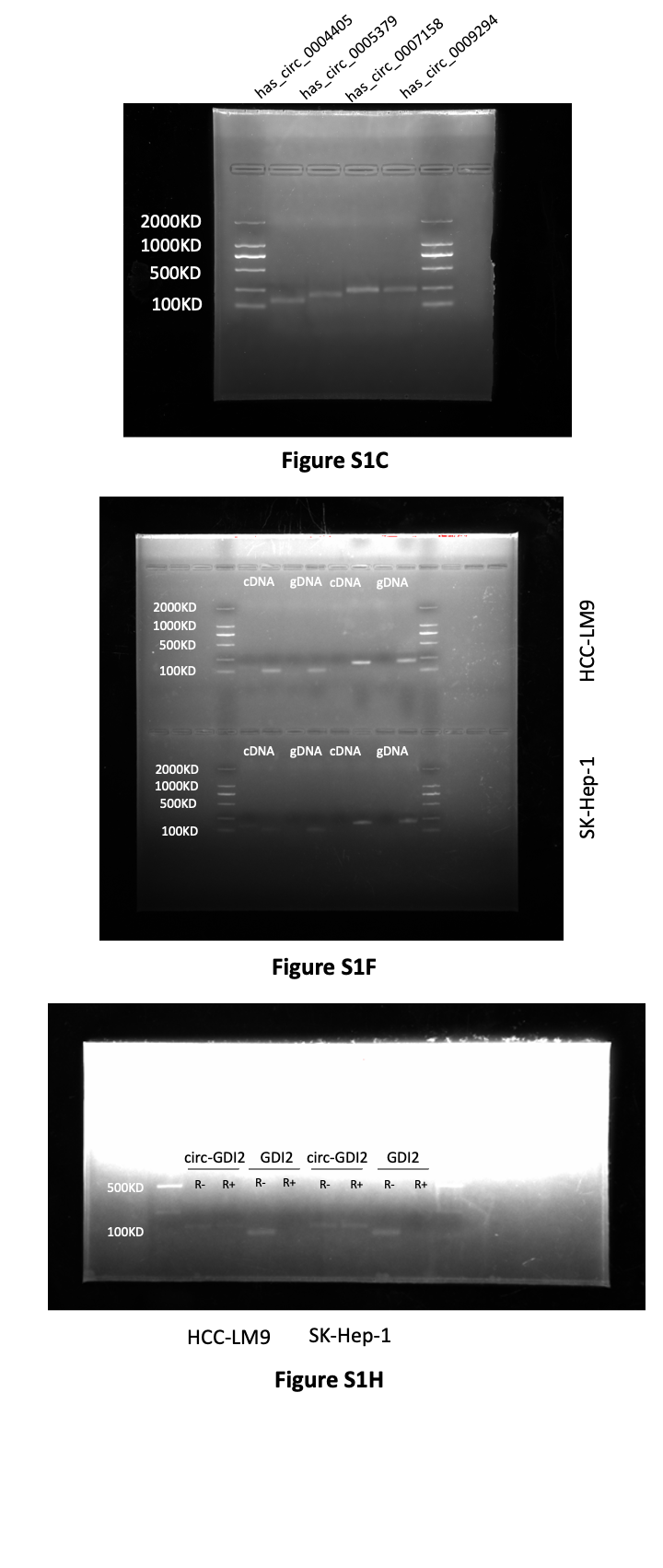


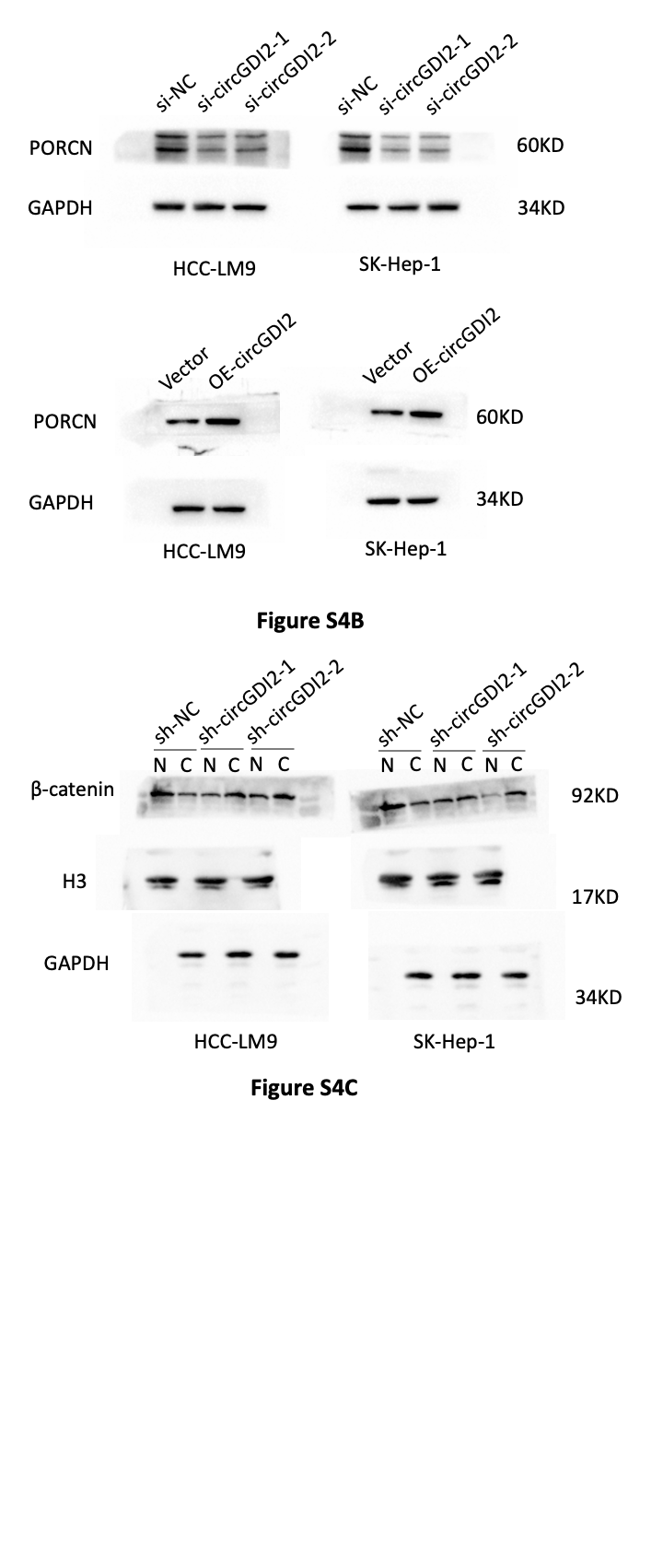


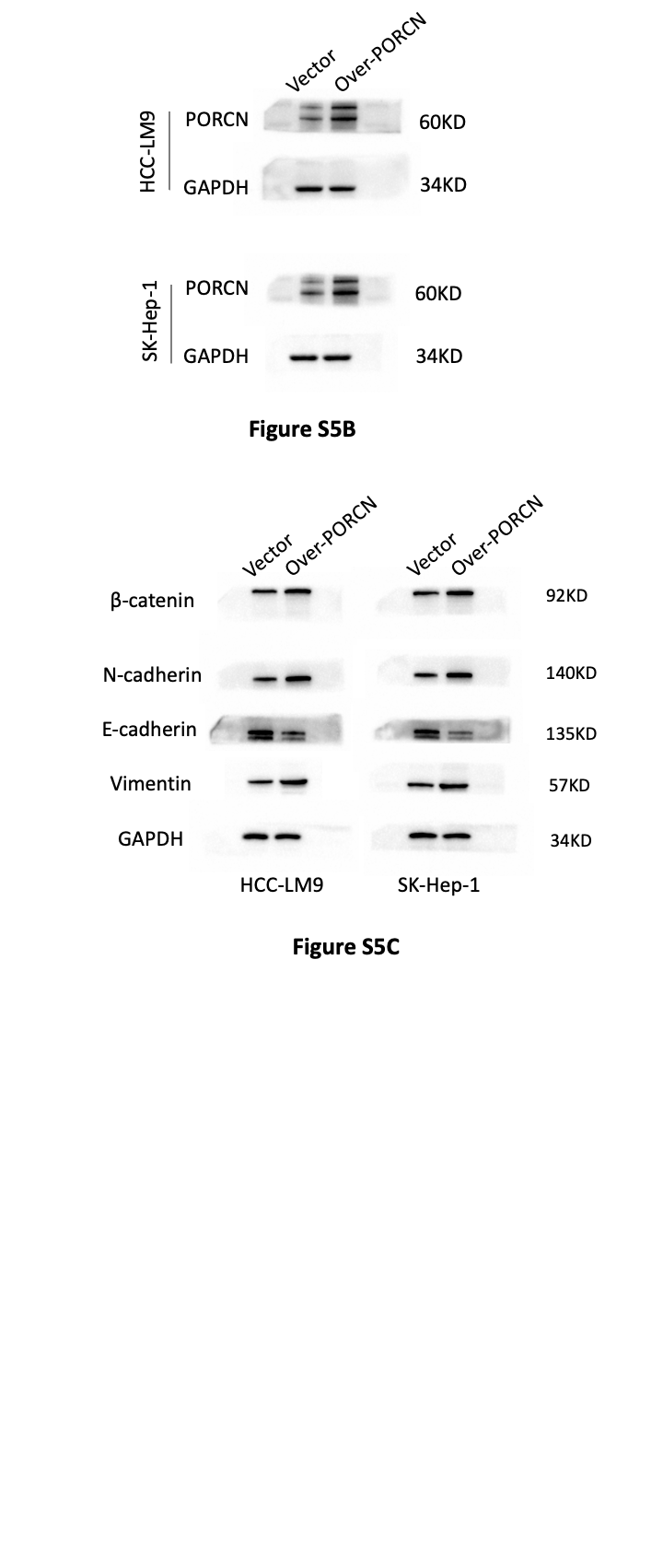


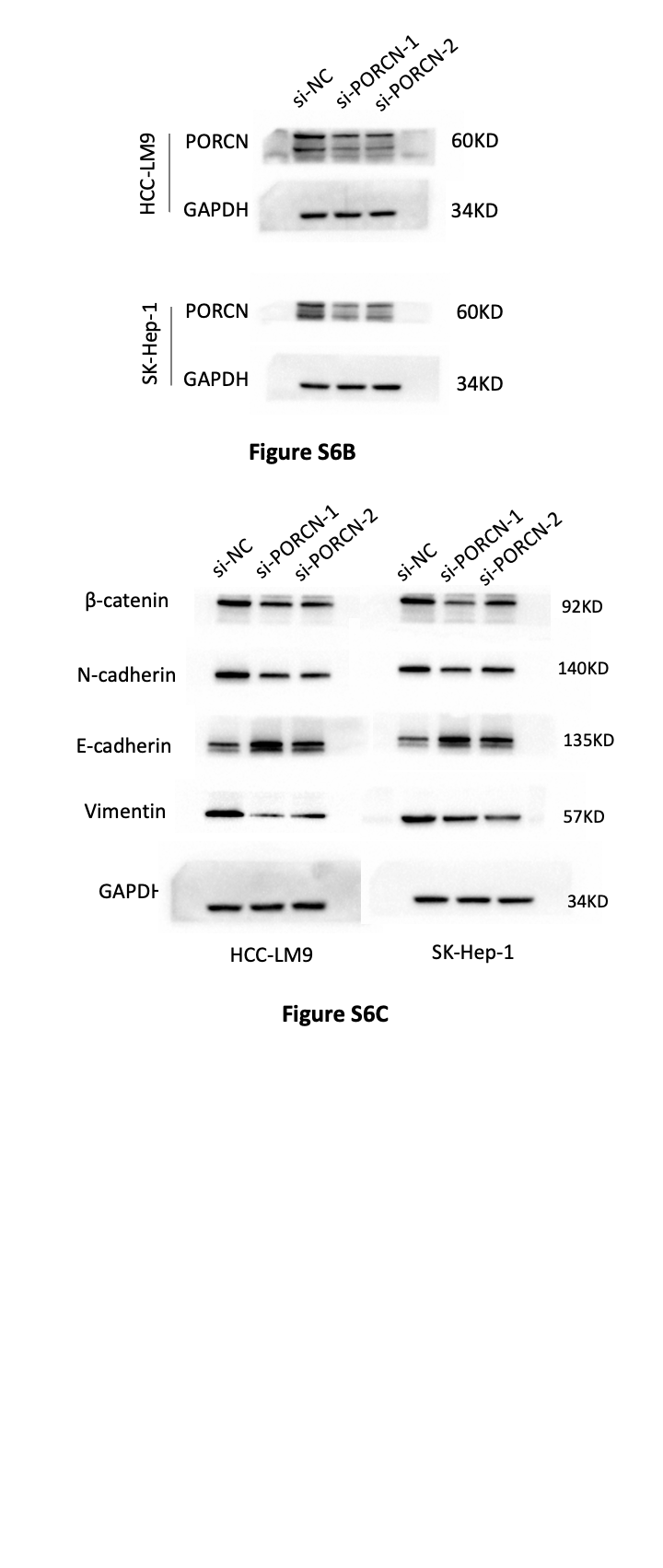


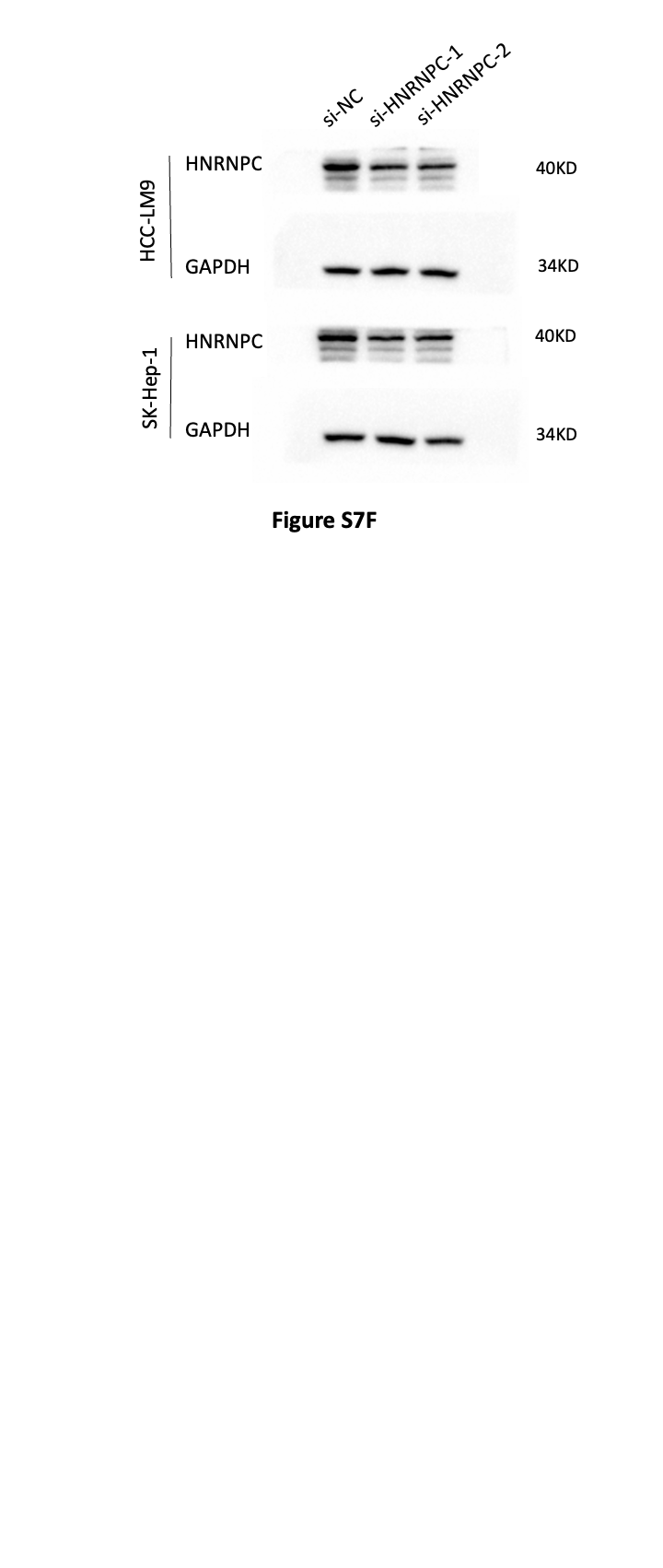


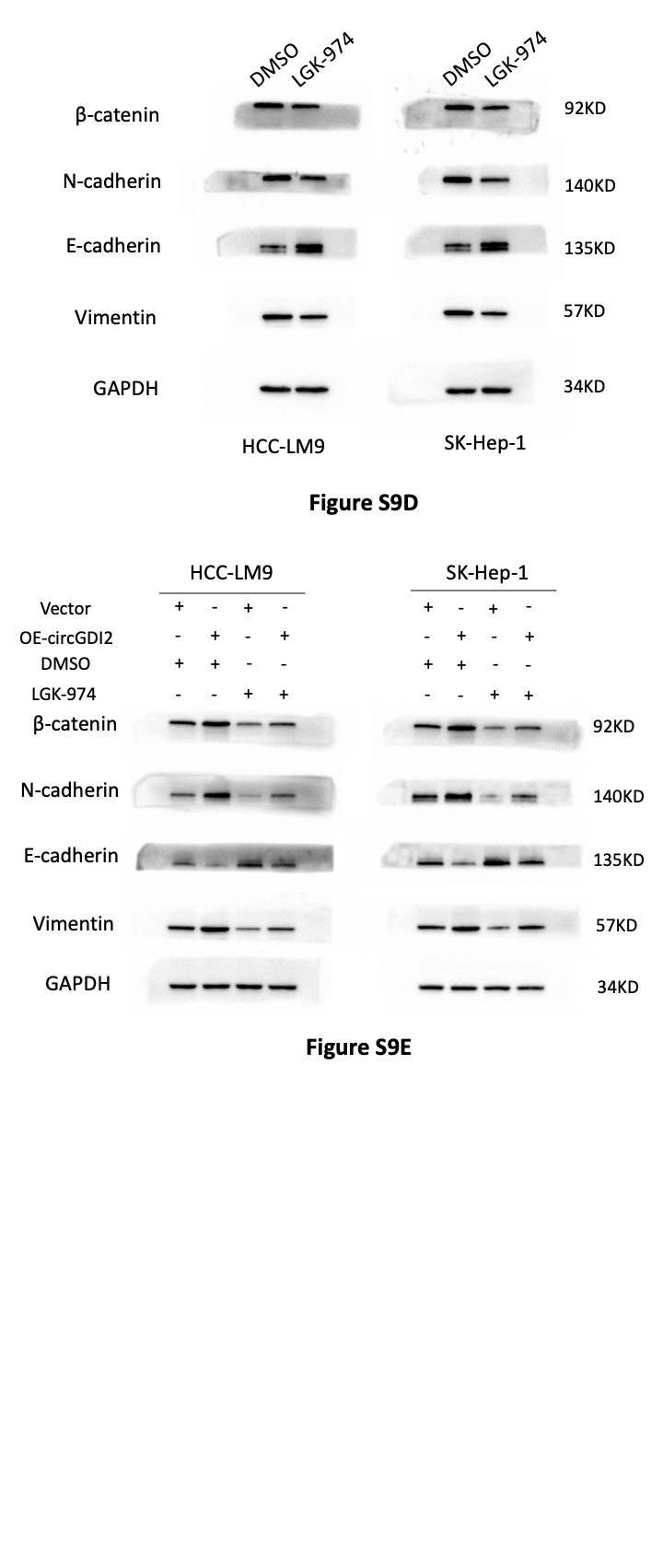

Supplement: Supplementary file 5 — Supplementary Material 5. [file 12943_2026_2638_MOESM5_ESM.docx]
