# Supplementary material for: Targeting circGDI2 disrupt HNRNPC-mediated mPORCN stabilization and enhance LGK-974 anti-tumor therapy in hepatocellular carcinoma
Source: Mol Cancer. 2026 Mar 10;25:110. doi: 10.1186/s12943-026-02638-1 (PMC13088843; doi:10.1186/s12943-026-02638-1)
Supplement: Supplementary file 7 — Supplementary Material 7. [file 12943_2026_2638_MOESM7_ESM.docx]

**Supplemental information includes:**

Supporting Figure S1–11

Tables S1–S3, S6, S9-S11

Other supplemental material for this manuscript includes:

Tables S4–S5, S7-S8, S12 (Excel format)

**Figure S1**

**
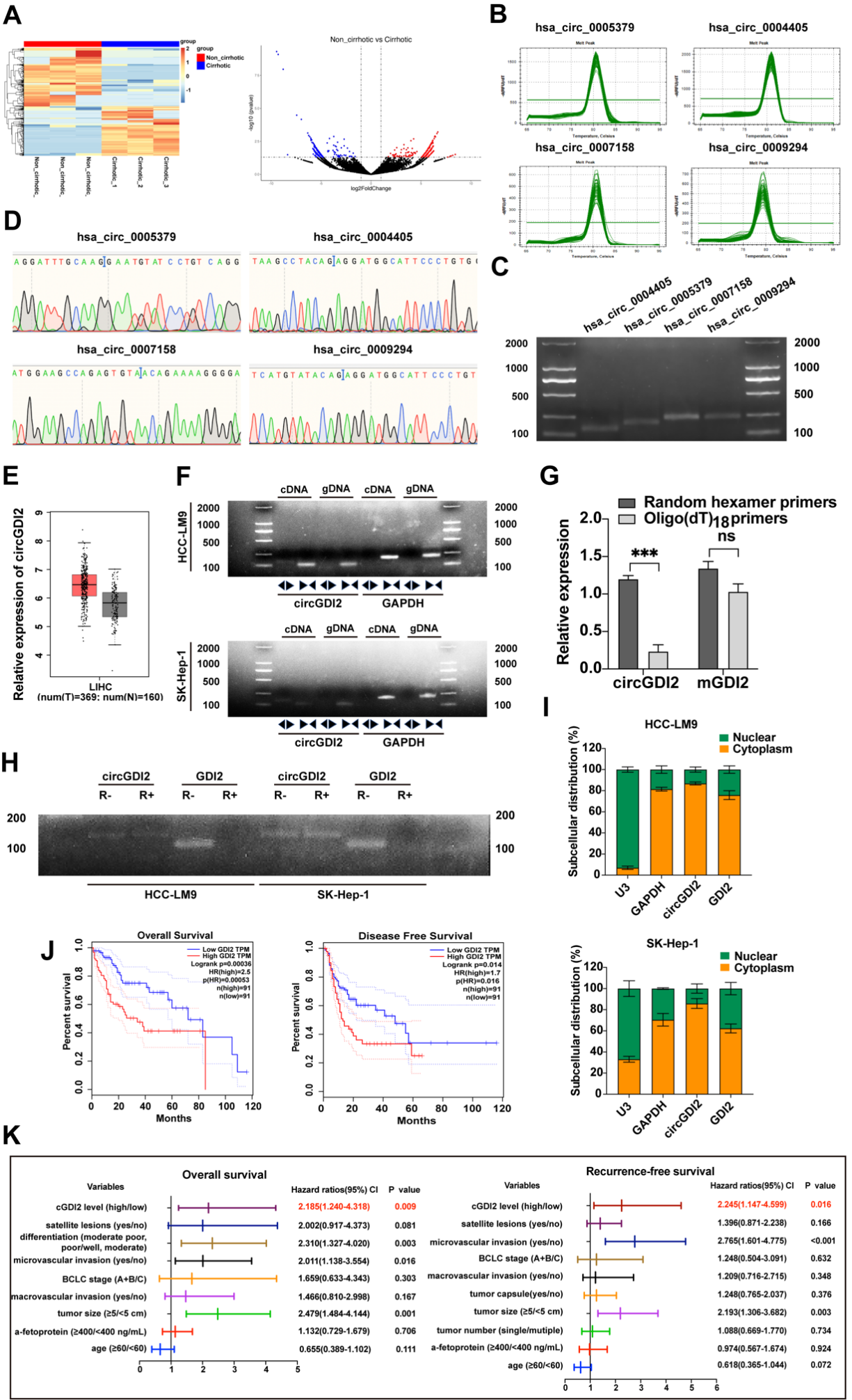
**

**Supporting figure S1:** (A) Heatmap (left) and volcano plot (right) representing sequencing results of cirrhotic HCC and non-cirrhotic HCC. (B) Melting curves of PCR products for 4 differentially expressed circRNAs. (C) Nucleic acid electrophoresis results of PCR products for the 4 differentially expressed circRNAs. (D) Sanger Sequencing of the 4 differentially expressed circRNAs showing the splice sites. (E) Comparison of GDI2 expression levels between HCC and adjacent normal tissues using the GEPIA database. (F) Structural characterization of circGDI2 in HCC cells was confirmed by designed forward and reverse primers with GAPDH as a negative control. (G) Expression of circGDI2 and mGDI2 in HCC cells were detected using random primers and Oligo(dT)18 primers. (H) Nucleic acid electrophoresis verified the expression levels of circGDI2 and GDI2 after RNase R management. (I) Subcellular localization of circGDI2 was detected by RNA nuclear-cytoplasmic fractionation assay. (J) Kaplan-Meier curves showing the OS (left) and RFS (right) of patients in the GEPIA database, stratified by *GDI2* expression levels. (K) Multivariate analyses showing hazard factors for OS (left) and RFS (right) of the matched cohort. HCC: hepatocellular carcinoma; PCR: polymerase chain reaction; GEPIA: Gene Expression Profiling Interactive Analysis; OS: overall survival; RFS: recurrence free-survival; Data are shown as mean ± SEM with p values by Mann-Whitney tests. ns: no significancy; ***: P<0.001.

**Figure S2**

**
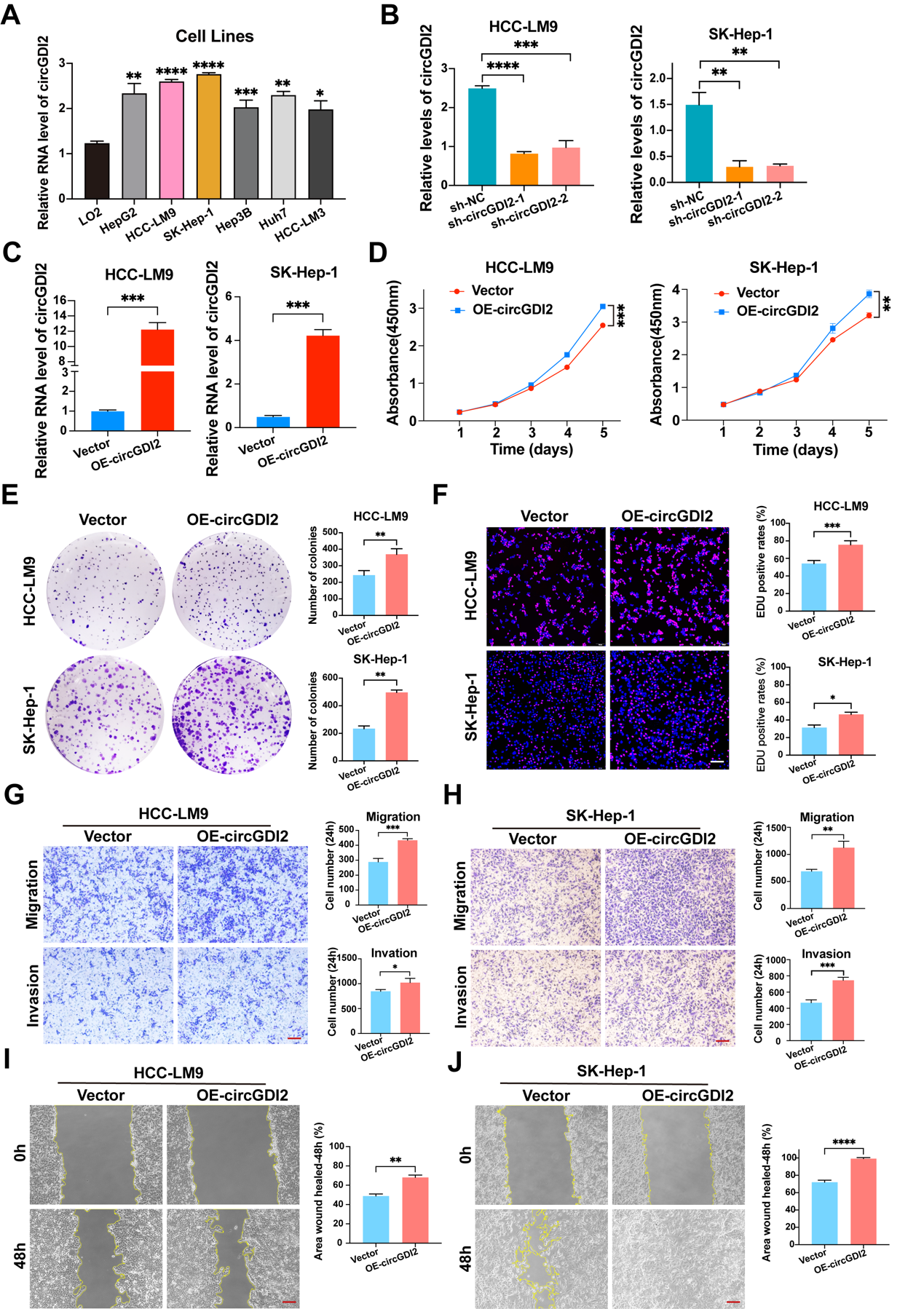
**

**Supporting figure S2:** (A) The expression levels of circGDI2 in hepatocyte LO2 and HCC cell lines. (B) Validation of circGDI2 levels in HCC cells transfected with sh-circGDI2 lentivirus by qRT-PCR. (C) Validation of circGDI2 levels in HCC cells transfected with OE-circGDI2 lentivirus by qRT-PCR. (D) CCK-8 assay was performed in HCC cells transfected with OE-circGDI2 lentivirus. (E) Colony formation assay was conducted in HCC cells transfected with OE-circGDI2 lentivirus. (F) EdU assay was performed to detect the effects of circGDI2 on the capacity of DNA duplication of HCC cells transfected with OE-circGDI2 lentivirus. (G-H) Transwell assay was performed in HCC cells transfected with OE-circGDI2 lentivirus to evaluate the migration capability. (I-J) Wound healing assay was performed in HCC cells transfected with OE-circGDI2 lentivirus. HCC: hepatocellular carcinoma; qRT-PCR: quantitative reverse transcription polymerase chain reaction; OE: overexpression; CCK-8: Cell Counting Kit-8; Data are shown as mean ± SEM with p values by Mann-Whitney tests. *P: <0.05; **P: <0.01; ***P: <0.001; ****: P<0.0001. Scale bars, 100μm.

**Figure S3**

**
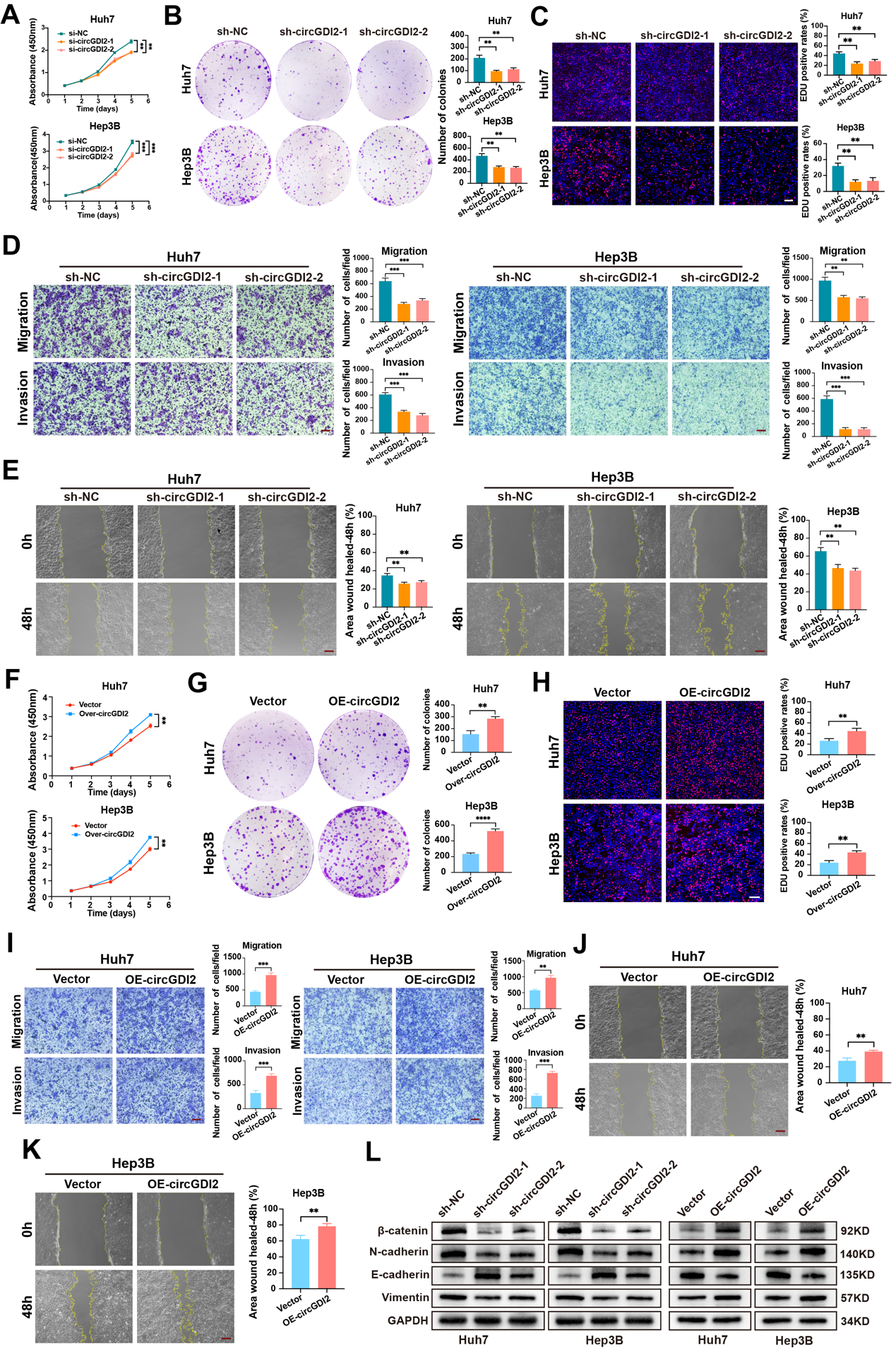
**

**Supporting figure S3:** (A) CCK-8 assay was performed in HCC cells transfected with si-circGDI2. (B) Colony formation assay was conducted in HCC cells transfected with sh-circGDI2. (C) EdU assay was performed to detect the effects of circGDI2 on the capacity of DNA duplication of HCC cells transfected with sh-circGDI2. (D) Transwell assay was performed in HCC cells transfected with sh-circGDI2 to evaluate the migration and invasion capability. (E) Wound healing assay was performed in HCC cells transfected with sh-circGDI2. (F) CCK-8 assay was performed in HCC cells transfected with OE-circGDI2 lentivirus. (G) Colony formation assay was conducted in HCC cells transfected with OE-circGDI2 lentivirus. (H) EdU assay was performed to detect the effects of circGDI2 on the capacity of DNA duplication of HCC cells transfected with OE-circGDI2 lentivirus. (I) Transwell assay was performed in HCC cells transfected with OE-circGDI2 lentivirus to evaluate the migration capability. (J-K) Wound healing assay was performed in HCC cells transfected with OE-circGDI2 lentivirus. (L) Western Blot showed the expression of EMT-related molecules in sh-circGDI2 and OE-circGDI2 HCC cells. CCK-8: Cell Counting Kit-8; HCC: hepatocellular carcinoma; EMT: Epithelial-Mesenchymal Transition; OE: overexpression; ns: no significancy; Data are shown as mean ± SEM with p values by Mann-Whitney tests. **P: <0.01; ***P: <0.001; ****: P<0.0001. Scale bars, 100μm.

**Figure S4**

**
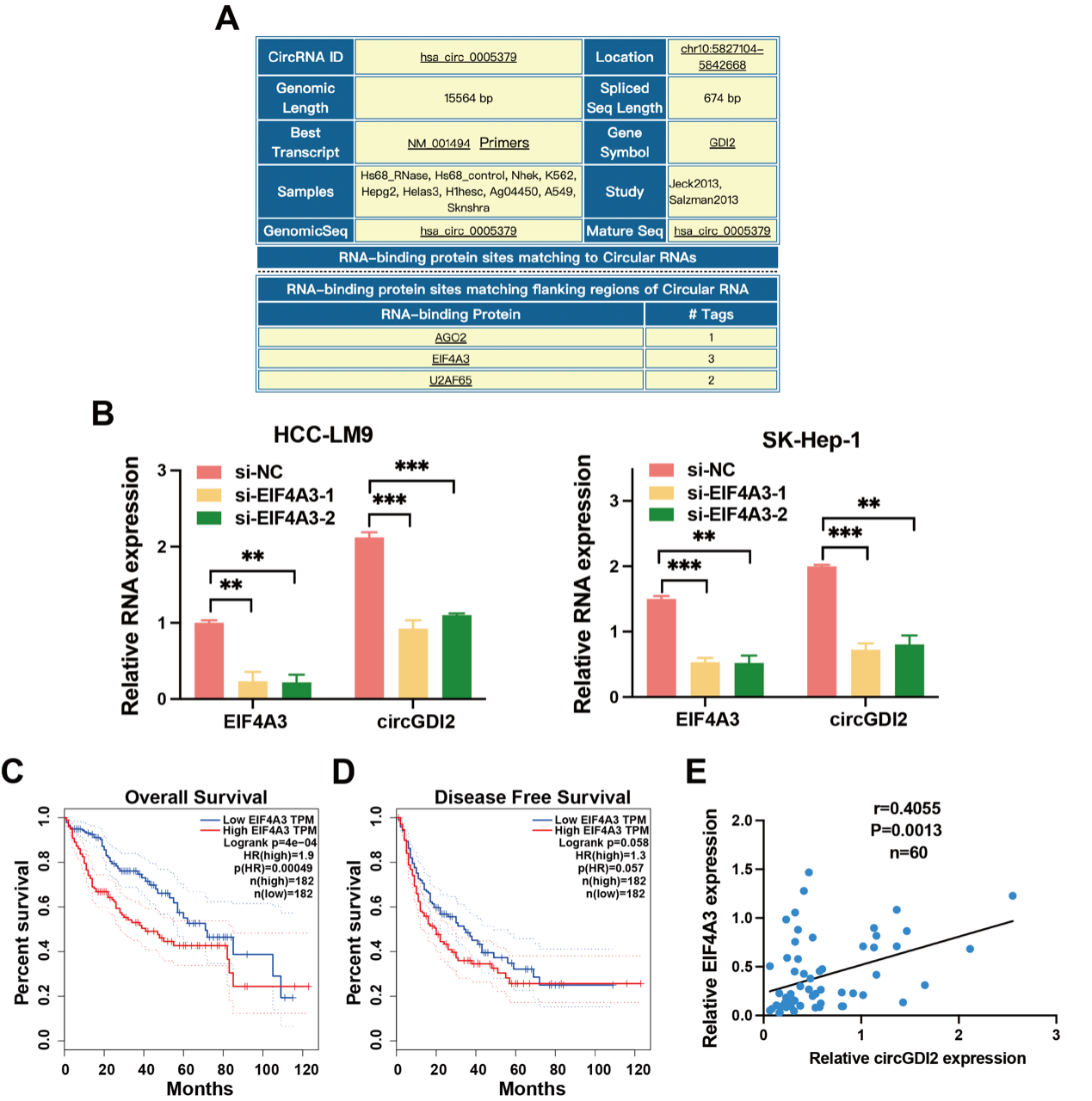
**

**Supporting figure S4:** (A) Putative RBPs in GDI2 pre-mRNA were predicted using the online CircInteractome database. (B) Relative levels of EIF4A3 and circGDI2 in HCC-LM9 and SK-Hep-1 cell transfected with EIF4A3 siRNAs. (C-D) The direct binding of TDP43 to the PSD3 pre-mRNA was confirmed using a RIP assay. (E) Pearson correlation analysis showing a positive correlation between EIF4A3 and circGDI2 in 60 HCC tissues. **P: <0.01; ***P: <0.001.

**Figure S5**

**
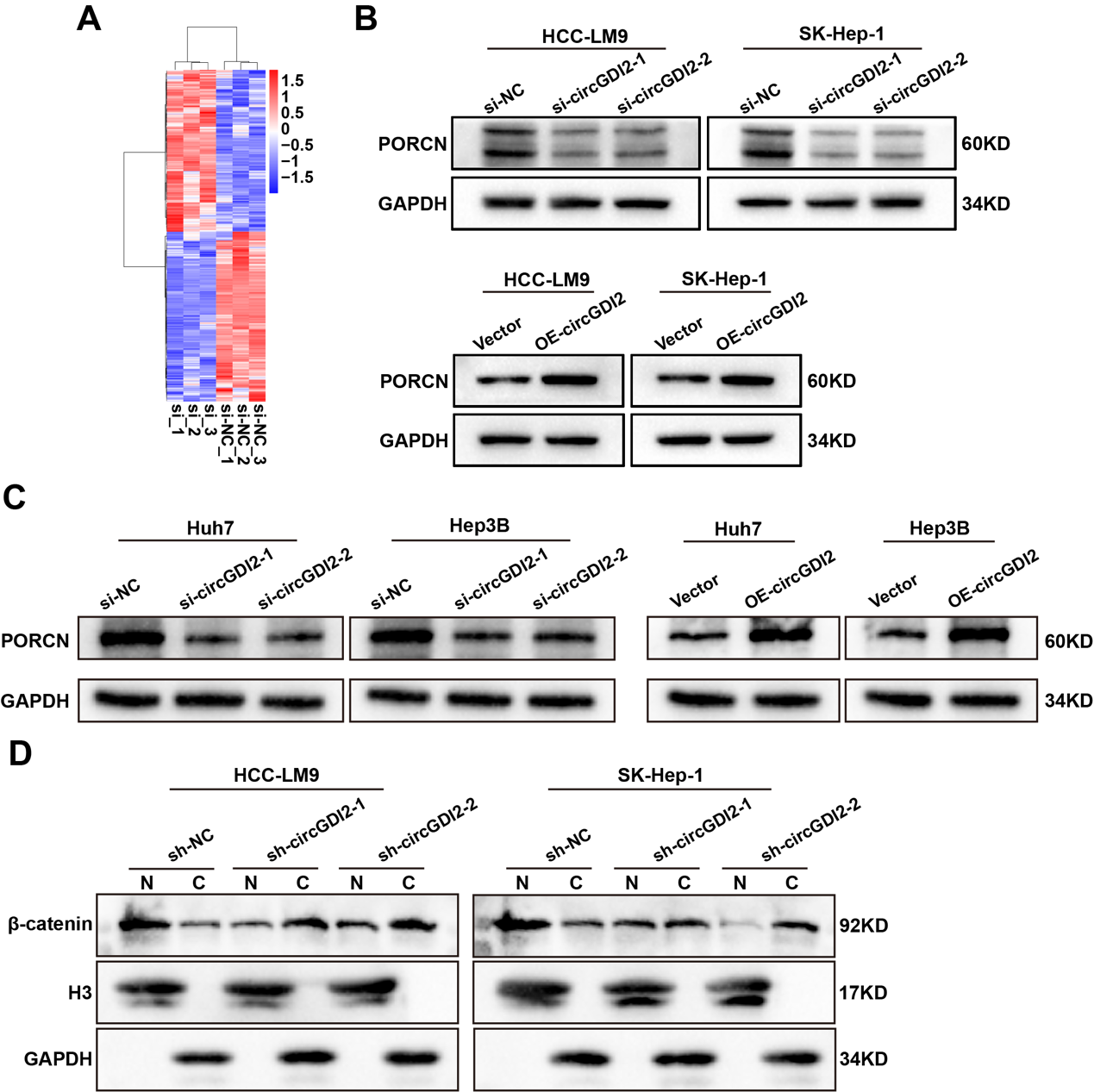
**

**Supporting figure S5:** (A) Heatmap representing sequencing results of HCC-LM9 cells transfected with si-NC and si-circGDI2. (B-C) Western Blot detected the expression of PORCN in HCC cells transfected with si-circGDI2 and OE-circGDI2. (D) Western blotting analysis of β-catenin expression in the nuclear (N) and cytoplasmic (C) fractions in HCC cells transfected with sh-circGDI2 and OE-circGDI2. HCC: hepatocellular carcinoma; OE: overexpression; *P: <0.05; **P: <0.01; ***P: <0.001.

**Figure S6**

**
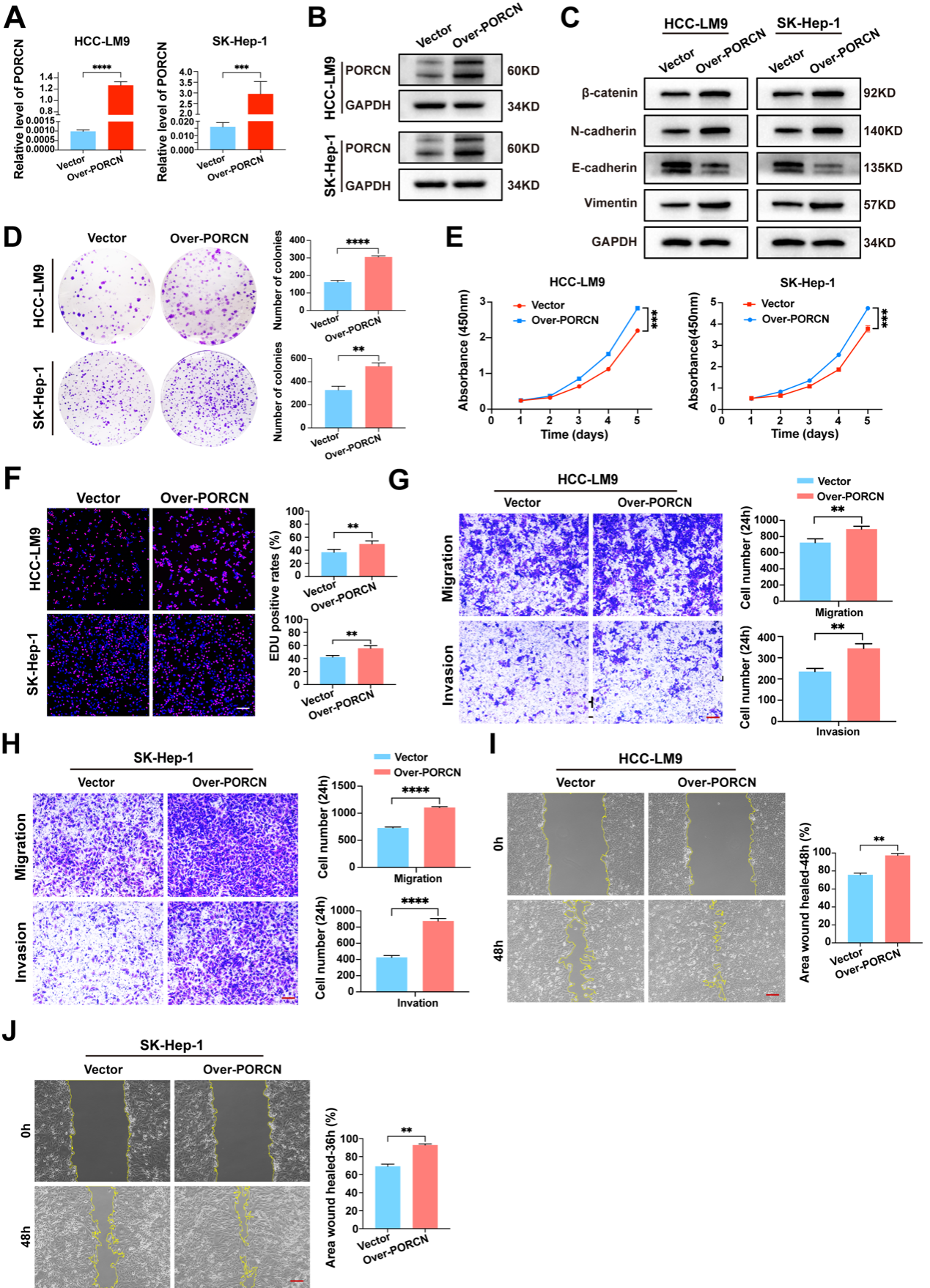
**

**Supporting figure S6:** (A) The expression levels of PORCN were measured by qRT-PCR after transfecting HCC cells with Over-PORCN. (B) Western blot verified the expression levels of PORCN after transfecting HCC cells with Over-PORCN. (C) Western Blot showed for the expression of EMT-related molecules in HCC cells transfected with Over-PORCN. (D) Colony formation assay was performed in HCC cells transfected with Over-PORCN (E) CCK-8 assay was conducted in HCC cells transfected with Over-PORCN. (F) EdU assay was performed to detect the capacity of DNA duplication of HCC cells transfected with Over-PORCN. (G-H) Transwell assay was performed in HCC cells transfected with Over-PORCN to evaluate the migration capability. (I-J) Wound healing assay was performed in HCC cells transfected with Over-PORCN. qRT-PCR: quantitative reverse transcription polymerase chain reaction; HCC: hepatocellular carcinoma; EMT: Epithelial-Mesenchymal Transition; CCK-8: Cell Counting Kit-8; Data are shown as mean ± SEM with p values by Mann-Whitney tests. **P: <0.01; ***P: <0.001; ****: P<0.0001. Scale bars, 100μm.

**Figure S7**

**
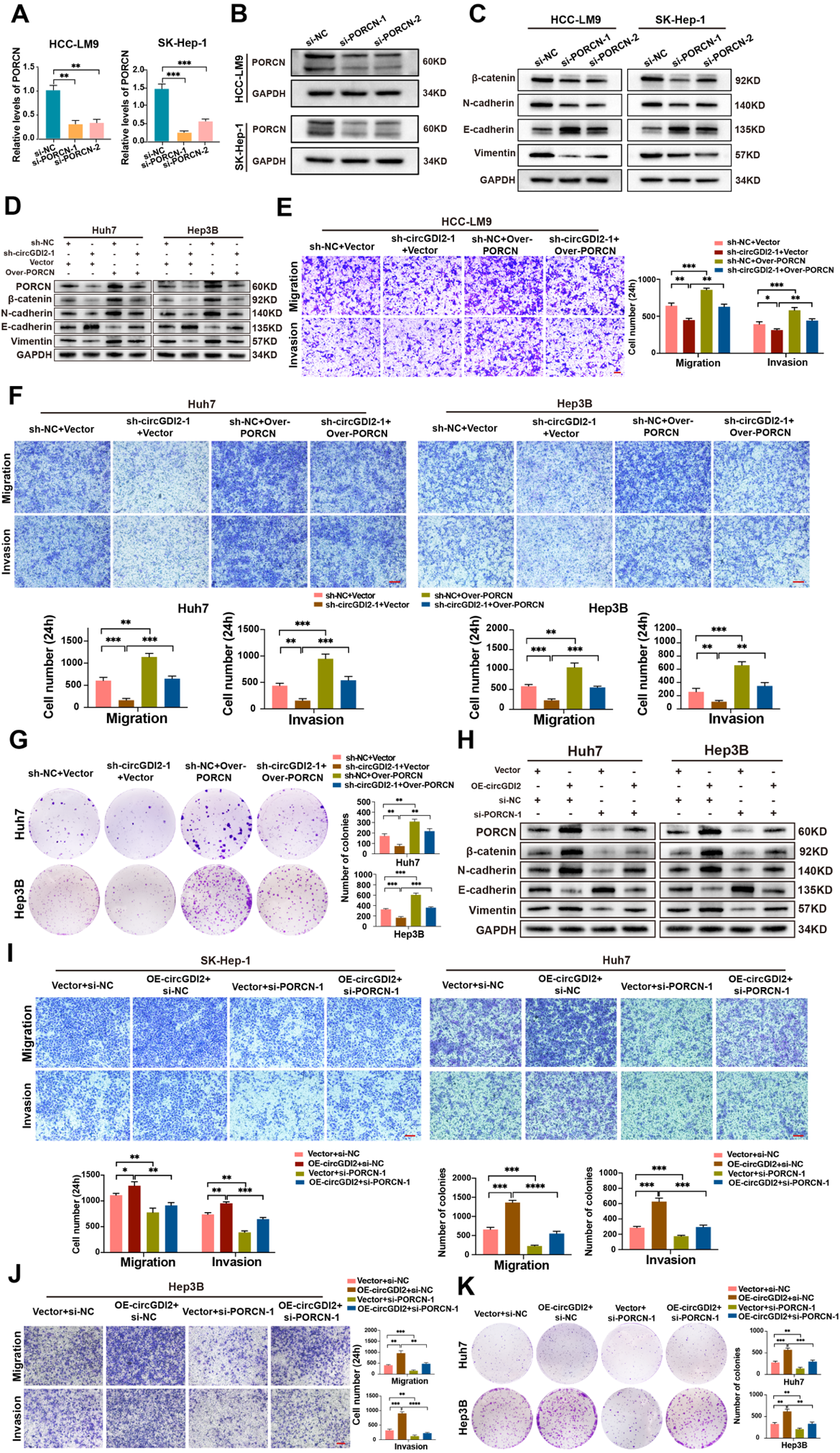
**

**Supporting figure S7:** (A) The expression levels of PORCN were measured by qRT-PCR after transfecting HCC cells with si-PORCN. (B) Western blot verified the expression levels of PORCN after transfecting HCC cells with si-PORCN. (C) Western Blot showed for the expression of EMT-related molecules in HCC cells transfected with si-PORCN. (D) Western Blot for the expression of EMT-related molecules in the indicated Huh7 and Hep3B cells. (E-F) Transwell assay showed the migration and invasion capacity of the indicated HCC cells. (G) Colony formation assay showed the proliferation capacity of the indicated HCC cells. (H) Western Blot for the expression of EMT-related molecules in the indicated Huh7 and Hep3B cells. (I-J) Transwell assay showed the migration and invasion capacity of the indicated HCC cells. (K) Colony formation assay showed the proliferation capacity of the indicated HCC cells. qRT-PCR: quantitative reverse transcription polymerase chain reaction; HCC: hepatocellular carcinoma; EMT: Epithelial-Mesenchymal Transition; Data are shown as mean ± SEM with p values by Mann-Whitney tests. *P: <0.05; **P: <0.01; ***P: <0.001; ****: P<0.000. Scale bars, 100μm.

**Figure S8**

**
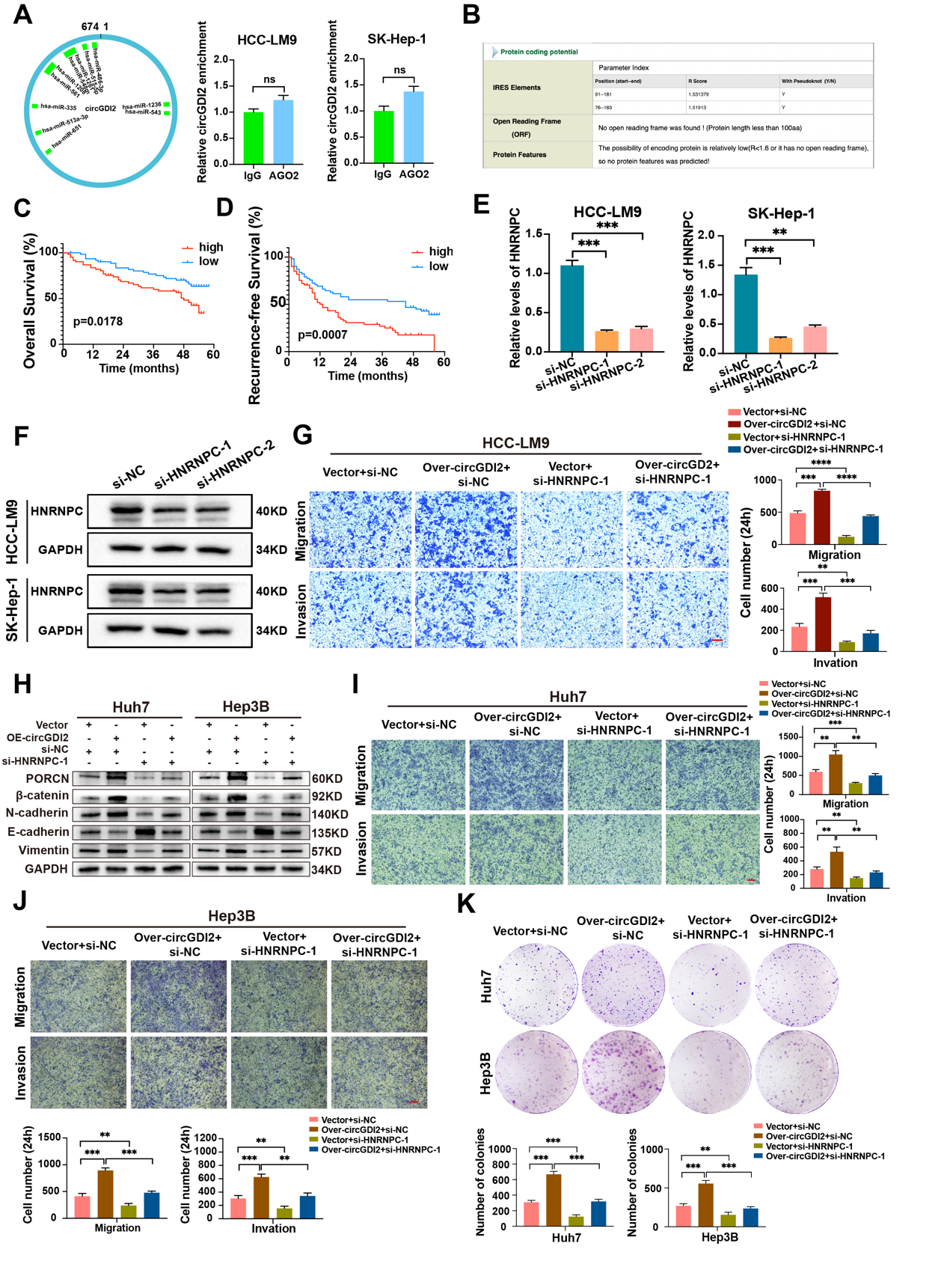
**

**Supporting figure S8:** (A) Predicted miRNAs that may bind to circGDI2, RIP assay didn’t enrich for circGDI2 in HCC cells using IgG and AGO2 antibodies. (B) The circRNADb database indicated a low coding potential for circGDI2. (C-D) Kaplan-Meier curves showing the OS (left) and RFS (right) of 123 HCC patients. Patients were stratified by the median expression levels of HNRNPC. (E) The expression levels of HNRNPC were measured by qRT-PCR after transfecting HCC cells with si-HNRNPC. (F) Western blot verified the expression levels of HNRNPC after transfecting HCC cells with si-HNRNPC. (G) Transwell assay showed the migration and invasion capacity of the indicated HCC-LM9 cells. (H) Western Blot for the expression of EMT-related molecules in the indicated Huh7 and Hep3B cells. (I-J) Transwell assay showed the migration and invasion capacity of the indicated HCC cells. (K) Colony formation assay showed the proliferation capacity of the indicated HCC cells. RIP: RNA immunoprecipitation; HCC: hepatocellular carcinoma; OS: overall survival; RFS: recurrence-free survival; qRT-PCR: quantitative reverse transcription polymerase chain reaction; ns: no significance; Data are shown as mean ± SEM with p values by Mann-Whitney tests or log-rank for Kaplan-Meier curves. **P: <0.01; ***P: <0.001; ****P: <0.0001. Scale bars, 100μm.

**Figure S9**

**
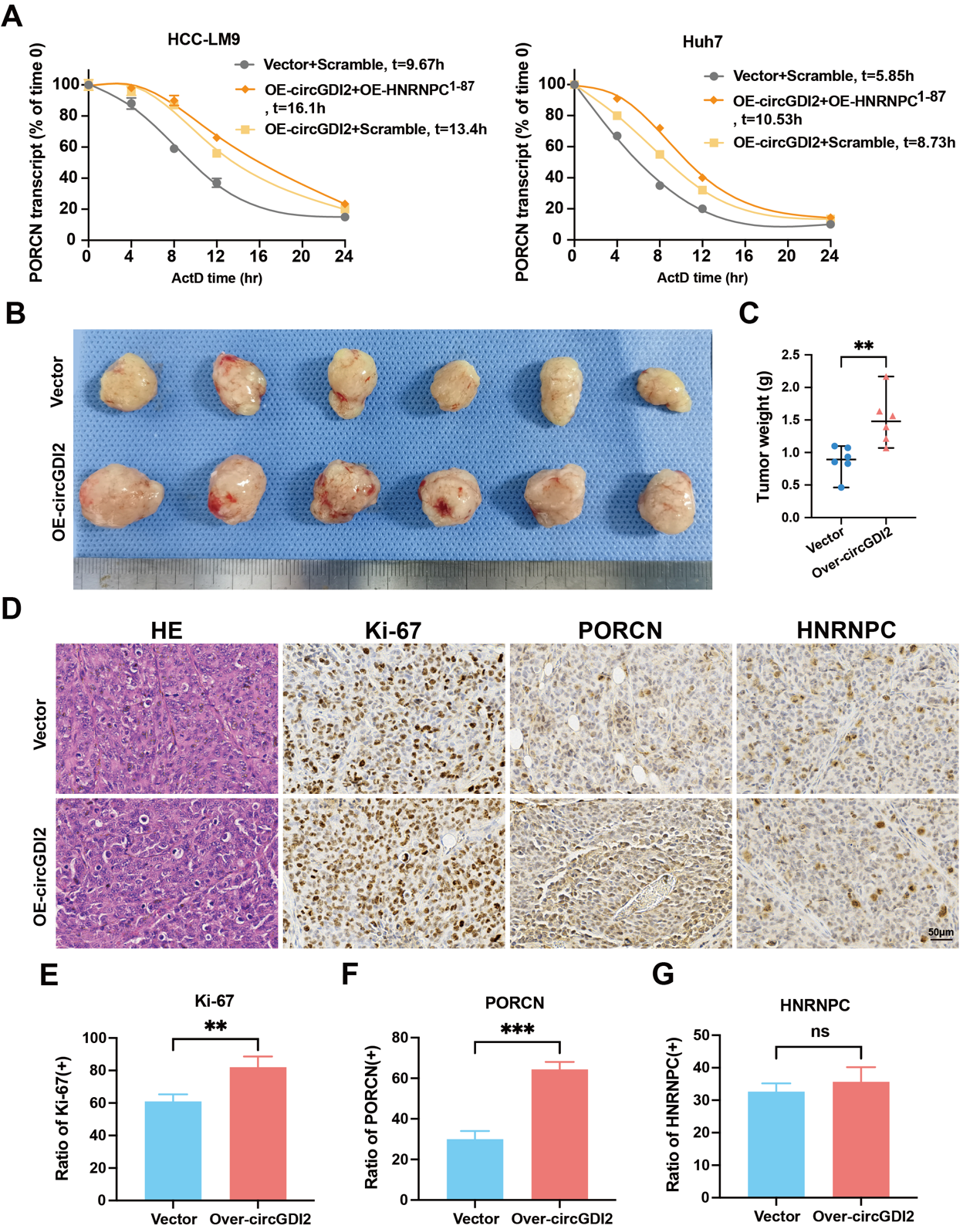
**

**Supplementary figure S9:** (A) The half-life of mPORCN after overexpressing the 1-87aa fragment of HNRNPC in both HCC-LM9 and Huh7 cell lines. (B) Subcutaneous xenograft tumors dissected from nude mice inoculated with indicated OE-circGDI2 HCC-LM9 cell. (C) Weight of subcutaneous xenograft tumors from figure A. (D) Representative HE, Ki-67, PORCN and HNRNPC staining images of subcutaneous xenograft tumors. (E-G) The proportion of IHC positive cells of subcutaneous xenograft tumors was indicated in the graph. HCC: hepatocellular carcinoma; HE: hematoxylin-eosin; ns: no significance; IHC: Immunohistochemistry. ns: no significance; **P: <0.01; ***P<0.001.

**Figure S10**

**
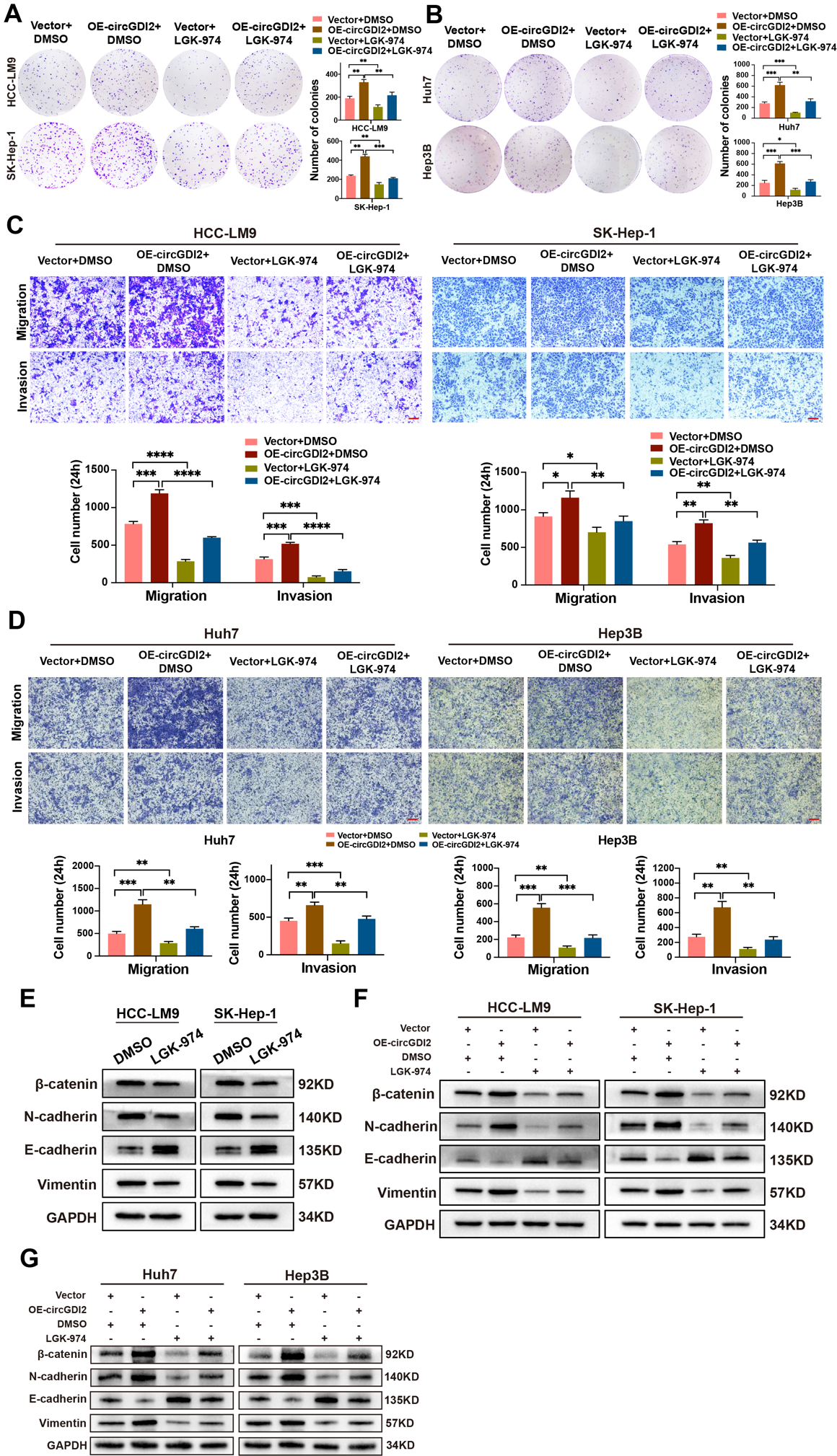
**

**Supporting figure S10:** (A-B) Colony formation assay showed the proliferation capacity of the indicated HCC cells. (C-D) Transwell assays showed the migration and invasion capacity of the indicated HCC cells. (E) Western Blot results for the expression of EMT-related molecules in HCC cells treated with LGK-974. (F-G) Western Blot results for the expression of EMT-related molecules in the indicated HCC cells. HCC: hepatocellular carcinoma; EMT: Epithelial-Mesenchymal Transition; DMSO: Dimethyl Sulfoxide; Data are shown as mean ± SEM with p values by Mann-Whitney tests. *P: <0.05; **P: <0.01; ***P: <0.001; ****: P<0.0001. Scale bars, 100μm.

**Figure S11**

**
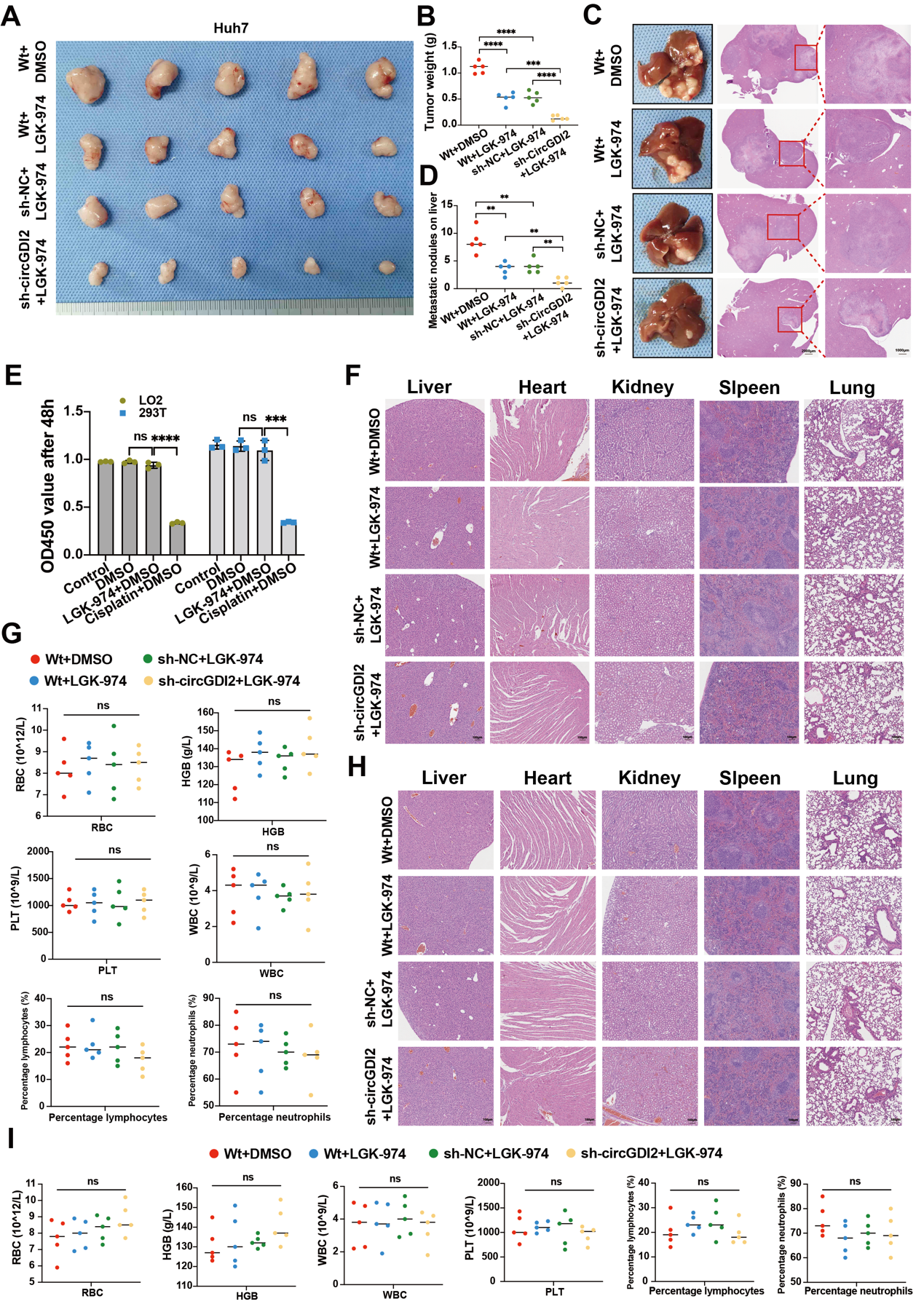
**

**Supporting figure S11:** (A) Representative images of subcutaneous xenografts with indicated Huh7 combined with LGK-974. (B) Tumors weight of subcutaneous xenografts was indicated in the dot graph. (C) Representative images of liver orthotopic-implantation models with indicated Huh7 combined with LGK-974. (D) The number of metastatic foci formed in the livers was indicated in the dot graph. (E) Evaluation of drug toxicity of LGK-974 in normal human cells. (F) Histological analysis of major organs among groups using HE staining. (G) Comparison of red blood cell (RBC), hemoglobin (HGB), platelet (PLT), white blood cell (WBC), lymphocyte and neutrophil levels in mice as indicated. (H) Histological analysis of major organs among groups using HE staining. (I) Comparison of red blood cell (RBC), hemoglobin (HGB), platelet (PLT), white blood cell (WBC), lymphocyte and neutrophil levels in mice as indicated. ns: no significance; **P: <0.01; ***P: <0.001; ****: P<0.0001. Scale bars, 100μm.

**Table S1**

| Supplemental table 1. The relationship between the expression level of hsa_circ_0005379 in cancer tissues and clinicopathological features in 123 HCC patients undergoing hepatectomy | | | |
| --- | --- | --- | --- |
| variables | Low group  (n=62) | High group  (n=61) | P value |
| Sex（male/female） | 55/7 | 50/11 | 0.290 |
| Age（≥60/＜60 year） | 19/43 | 19/42 | 0.952 |
| HBV-DNA（≥103/< 103 copies/mL) | 18/44 | 21/40 | 0.520 |
| AFP（≥400/<400 ng/mL) | 17/45 | 28/33 | 0.033 |
| Tumor number（single/multiple） | 15/47 | 20/41 | 0.291 |
| Tumor size（≥5/<5 cm) | 22/40 | 31/30 | 0.086 |
| Tumor capsule（Yes/No） | 33/29 | 39/22 | 0.228 |
| Macrovascular invasion（Yes/No） | 3/59 | 12/49 | 0.012 |
| BCLC stage（A+B/C stage） | 5/57 | 15/46 | 0.013 |
| MVI（Yes/No） | 16/46 | 29/32 | 0.012 |
| Differentiation degree（Moderate-Low, Low/High, Moderate） | 23/39 | 39/22 | 0.003 |
| Liver cirrhosis（Yes/No） | 34/28 | 36/25 | 0.640 |
| Satellite lesions（Yes/No） | 3/59 | 6/55 | 0.323 |
| HBV-DNA: Hepatitis B Virus-Deoxyribonucleic Acid; AFP: α-fetoprotein; BCLC: Barcelona Clinic Liver Cancer | | | |

**Table S2**

| Supplemental table 2 Univariate COX proportional hazards regression analysis of OS and RFS risk factors | | | | | | | |
| --- | --- | --- | --- | --- | --- | --- | --- |
| variables | OS | | |  | RFS | | |
|  | HR | 95% CI | P value |  | HR | 95% CI | P value |
| Sex（male） | 0.958 | 0.452-2.031 | 0.911 |  | 0.873 | 0.483-1.578 | 0.653 |
| Age（≥60 year） | 0.591 | 0.316-1.103 | 0.099 |  | 0.609 | 0.376-0.988 | 0.045 |
| HBV-DNA（≥103 copies/mL) | 1.306 | 0.751-2.270 | 0.344 |  | 1.219 | 0.774-1.920 | 0.394 |
| AFP（≥400 ng/mL) | 2.364 | 1.383-4.041 | 0.002 |  | 2.372 | 1.530-3.676 | <0.001 |
| Tumor number（multiple） | 1.511 | 0.855-2.672 | 0.155 |  | 1.699 | 1.080-2.671 | 0.022 |
| Tumor size（≥5 cm) | 2.228 | 1.297-3.828 | 0.004 |  | 3.022 | 1.933-4.724 | <0.001 |
| Tumor capsule（Yes） | 1.288 | 0.752-2.206 | 0.356 |  | 1.549 | 1.007-2.380 | 0.046 |
| Macrovascular invasion（Yes） | 2.559 | 1.310-4.998 | 0.006 |  | 2.480 | 1.404-4.381 | 0.002 |
| BCLC stage（C stage） | 2.874 | 1.573-5.250 | 0.001 |  | 2.230 | 1.314-3.787 | 0.003 |
| MVI（Yes） | 3.256 | 1.897-5.589 | <0.001 |  | 3.758 | 2.393-5.904 | <0.001 |
| Differentiation degree（Moderate-Low, Low） | 3.284 | 1.826-5.909 | <0.001 |  | 1.314 | 0.876-3.287 | 0.102 |
| Liver cirrhosis（Yes） | 1.314 | 0.756-2.283 | 0.333 |  | 0.994 | 0.645-1.533 | 0.979 |
| Satellite lesions（Yes） | 2.778 | 1.181-6.534 | 0.019 |  | 2.415 | 1.159-5.035 | 0.019 |
| CircGDI2 expression（high） | 1.985 | 1.141-4.452 | 0.015 |  | 1.645 | 1.065-2.540 | 0.025 |
| OS, overall survival; RFS: recurrence free survival; HR, hazard; CI, confidence interval; HBV-DNA: Hepatitis B Virus-Deoxyribonucleic Acid; AFP: α-fetoprotein; BCLC: Barcelona Clinic Liver Cancer; MVI, microvascular invasion | | | | | | | |

**Table S3**

| Supplemental table 3. Multivariate COX proportional hazards regression analysis of OS and RFS risk factors | | | | | | | |
| --- | --- | --- | --- | --- | --- | --- | --- |
| variables | OS | | |  | RFS | | |
|  | HR | 95% CI | P value |  | HR | 95% CI | P value |
| Age（≥60 year） | 0.655 | 0.389-1.102 | 0.111 |  | 0.618 | 0.365-1.044 | 0.072 |
| AFP（≥400 ng/mL) | 1.132 | 0.729-1.679 | 0.706 |  | 0.974 | 0.567-1.674 | 0.924 |
| Tumor number（multiple） |  |  |  |  | 1.088 | 0.669-1.770 | 0.734 |
| Tumor size（≥5 cm) | 2.479 | 1.484-4.144 | 0.001 |  | 2.193 | 1.306-3.682 | 0.003 |
| Tumor capsule（Yes） |  |  |  |  | 1.248 | 0.765-2.037 | 0.376 |
| Macrovascular invasion（Yes） | 1.466 | 0.810-2.998 | 0.167 |  | 1.209 | 0.716-2.715 | 0.348 |
| BCLC stage（C stage） | 1.659 | 0.633-4.343 | 0.303 |  | 1.248 | 0.504-3.091 | 0.632 |
| MVI（Yes） | 2.011 | 1.138-3.554 | 0.016 |  | 2.765 | 1.601-4.775 | <0.001 |
| Differentiation degree（Moderate-Low, Low） | 2.310 | 1.327-4.020 | 0.003 |  |  |  |  |
| Satellite lesions（Yes） | 2.002 | 0.917-4.373 | 0.081 |  | 1.396 | 0.871-2.238 | 0.166 |
| CircGDI2 expression（high） | 2.185 | 1.240-4.318 | 0.009 |  | 2.245 | 1.147-4.599 | 0.016 |

OS, overall survival; RFS, recurrence free survival; HR, hazard; CI, confidence interval; HBV-DNA: Hepatitis B Virus-Deoxyribonucleic Acid; AFP: α-fetoprotein; BCLC: Barcelona Clinic Liver Cancer; MVI, microvascular invasion

**Table S6. RBPs potentially binding circGDI2 predicted from circAltas.**

| Uniform ID | RBP | #binding sites in upstream flanking sequences | #binding sites in downstream flanking sequences | #binding sites in cirexons |
| --- | --- | --- | --- | --- |
| circGDI2(L7).1 | BCLAF1 | 0 | 0 | 3 |
| circGDI2(L7).1 | YBX3 | 0 | 0 | 1 |
| circGDI2(L7).1 | PUM2 | 0 | 0 | 1 |
| circGDI2(L7).1 | CPSF7 | 1 | 0 | 1 |
| circGDI2(L7).1 | CPSF6 | 0 | 0 | 1 |
| circGDI2(L7).1 | NIPBL | 0 | 0 | 1 |
| circGDI2(L7).1 | UPF1 | 2 | 0 | 2 |
| circGDI2(L7).1 | AGO2 | 4 | 0 | 5 |
| circGDI2(L7).1 | IGF2BP1 | 0 | 0 | 1 |
| circGDI2(L7).1 | CSTF2T | 4 | 0 | 1 |
| circGDI2(L7).1 | WDR33 | 0 | 0 | 1 |
| circGDI2(L7).1 | HNRNPC | 66 | 0 | 4 |

**Table S9. Information of siRNAs used in this study**

| **Name** | **Target sequence** | **Supplier** |
| --- | --- | --- |
| si-circGDI2-1 | CAAGGAATGTATCCTGTCA | Ribobio |
| si-circGDI2-2 | GGATTTGCAAGGAATGTAT | Ribobio |
| si-HNRNPC-1 | CTCGAAACGTCAGCGTGTA | Ribobio |
| si-HNRNPC-2 | GCCTTCGTTCAGTATGTTA | Ribobio |
| si-PORCN-1 | AAGTTGTCACAAGCTGGAACC | Ribobio |
| si-PORCN-2 | AAGAGACCGACUCAUUAGCTT | Ribobio |
| si-NC | CCTAAGGTTAAGTCGCCCTCG | Ribobio |

shRNAs’ sequencing is derived from the corresponding siRNA.

**Table S10. Primers used in this study**

| Name | Sequence (5’-3’) |
| --- | --- |
| Hsa_circ_0005379-Foward | TATGGCAAAAGCCCATACCT |
| Hsa_circ_0005379-Reverse | CCGTAGTAAGGGTTTCGATCC |
| Hsa_circ_0004405-Foward | TCTTGGGGAGAGAGTGGTTC |
| Hsa_circ_0004405-Reverse | CCAGCATATCCACAGGGAAT |
| Hsa_circ_0007158-Foward | GCTTGCGGTATCCACTGTCT |
| Hsa_circ_0007158-Reverse | CTGGGTCTGATCTCCACCAT |
| Hsa_circ_0007294-Foward | TCTCCTTCCCAAAAGACCAA |
| Hsa_circ_0007294-Reverse | CATGAAGTGCACTCCCCTTT |
| GDI2-Foward | CCAACTCCTGCCAGATCATT |
| GDI2-Reverse | TCCCTTGTGCTGCTACATTG |
| PORCN-Foward | AGCCGCCAGGAATTTTTCCA |
| PORCN-Reverse | AGCACGAGGTAGCACAGGA |
| GAPDH-Foward | ACTCCTCCACCTTTGACGC |
| GAPDH-Reverse | GCTGTAGCCAAATTCGTTGTC |
| Divergent GAPDH-Foward | GGCCTCCAAGGAGTAAGA |
| Divergent GAPDH-Reverse | GCCCAATACGACCAAATCA |
| β-catenin-Foward | AAAGCGGCTGTTAGTCACTGG |
| β-catenin-Reverse | CGAGTCATTGCATACTGTCCAT |
| HNRNPC-Foward | GATATTAACCTGGCTGCAGAG |
| HNRNPC-Reverse | TGATACACGCTGAGTAGAGG |
| U6-Foward | CTCGCTTCGGCAGCACA |
| U6-Reverse | AACGCTTCACGAATTTGCG |
| U3-forward | TTCTCTGAGCGTGTAGAGCACCGA |
| U3-reverse | GATCATCAATGGCTGACGGCAGTT |

**Table S11. Antibody used in this study**

| **Name** | **Host** | **Supplier** |
| --- | --- | --- |
| PORCN | Rabbit | ABclonal |
| HNRNPC | Rabbit | ABclonal |
| β-catenin | Rabbit | ABclonal |
| E-cadherin | Rabbit | ABclonal |
| N-cadherin | Rabbit | ABclonal |
| Vimentin | Rabbit | ABclonal |
| Ki-67 | Rabbit | Santa |
| IgG | Rabbit | Millipore |
| GAPDH | Rabbit | ZEN-BIOSCIENCE |
